# Origin of Irreversibility of Cell Cycle Start in Budding Yeast

**DOI:** 10.1371/journal.pbio.1000284

**Published:** 2010-01-19

**Authors:** Gilles Charvin, Catherine Oikonomou, Eric D. Siggia, Frederick R. Cross

**Affiliations:** 1 Laboratoire Joliot-Curie, Ecole Normale Supérieure, Lyon, France; 2 Laboratoire de Physique, Ecole Normale Supérieure, Lyon, France; 3 Université de Lyon, Lyon, France; 4 Center for Studies in Physics and Biology, The Rockefeller University, New York, New York, United States of America; 5 Laboratory of Yeast Molecular Genetics, The Rockefeller University, New York, New York, United States of America; Stanford University, United States of America

## Abstract

Budding yeast cells irreversibly commit to a new division cycle at a regulatory transition called Start. This essential decision-making step involves the activation of the SBF/MBF transcription factors. SBF/MBF promote expression of the G1 cyclins encoded by *CLN1* and *CLN2*. Cln1,2 can activate their own expression by inactivating the Whi5 repressor of SBF/MBF. The resulting transcriptional positive feedback provides an appealing, but as yet unproven, candidate for generating irreversibility of Start. Here, we investigate the logic of the Start regulatory module by quantitative single-cell time-lapse microscopy, using strains in which expression of key regulators is efficiently controlled by changes of inducers in a microfluidic chamber. We show that Start activation is ultrasensitive to G1 cyclin. In the absence of *CLN1,2*-dependent positive feedback, we observe that Start transit is reversible, due to reactivation of the Whi5 transcriptional repressor. Introduction of the positive feedback loop makes Whi5 inactivation and Start activation irreversible, which therefore guarantees unidirectional entry into S phase. A simple mathematical model to describe G1 cyclin turn on at Start, entirely constrained by empirically measured parameters, shows that the experimentally measured ultrasensitivity and transcriptional positive feedback are necessary and sufficient dynamical characteristics to make the Start transition a bistable and irreversible switch. Our study thus demonstrates that Start irreversibility is a property that arises from the architecture of the system (Whi5/SBF/Cln2 loop), rather than the consequence of the regulation of a single component (e.g., irreversible protein degradation).

## Abstract 2

In budding yeast, the commitment to entry into a new cell division cycle is made irreversible by positive feedback-driven expression of the G1 cyclins Cln1,2.

**Authors Summary**

In eukaryotes, the cell division cycle is composed of a tightly controlled sequence of well-defined steps, including duplication of the genetic material (DNA replication) and separation of chromosomes (mitosis). Entering a new round of division is a critical decision that depends on the cell's proper evaluation of the extracellular environment as well as of intracellular physiology. This commitment, once made, means the new cycle of division cycle cannot be aborted and must be successfully completed. Thanks to extensive research during the last three decades, the genetic mechanisms that govern entry into a new cell division cycle are well defined. However, it has remained mysterious how the regulatory network involved in cell cycle commitment could lead to a sharp, all-or-nothing decision-making process. In this study, we demonstrate that, in budding yeast, transcriptional positive feedback—a regulatory system in which a protein promotes transcription of its own gene—of the G1 cyclins Cln1,2 is the critical determinant for irreversible entry into a new cell cycle. This study thus bridges an important gap between the genetic architecture of a regulatory network and the functional requirement for robust unidirectional cell cycle transitions.

## Introduction

The Start transition is a key event in the yeast cell cycle during which the cell commits to a new round of division [1]. Start was originally defined as the point at which a yeast cell acquires resistance to mating pheromone [2]; in the presence of pheromone, post-Start cells proceed through the cell cycle to completion, whereas pre-Start cells arrest in an unbudded state. Start has therefore been described as a critical decision-making point at the end of G1, leading to an irreversible sequence of events.

**Figure 1:**
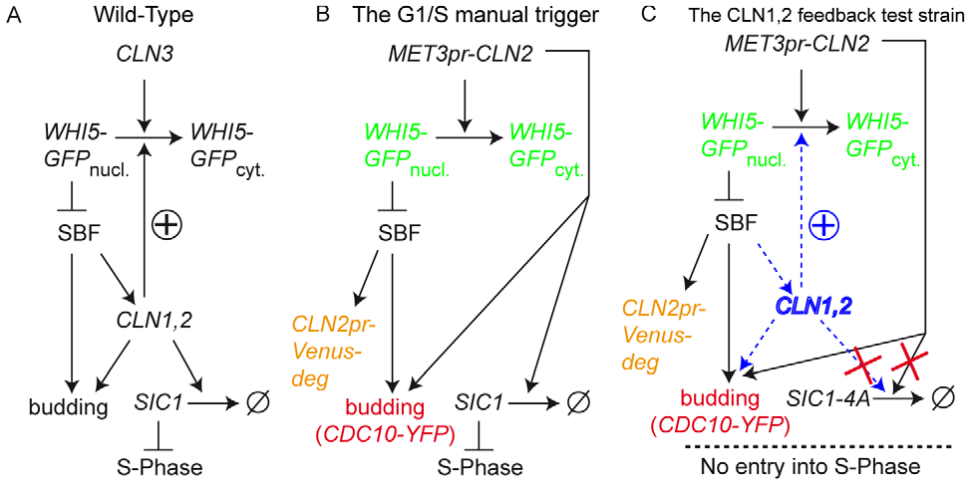
The Start transition network. (A) Schematic of the gene network involved in the budding yeast Start transition. See text for detailed description. (B) The “manual trigger” configuration used to measure nonlinearity in Start activation. Endogenous *CLN1,2,3* genes have been deleted. A copy of *CLN2* driven by the regulatable *MET3* promoter is used to trigger the Start transition. Colored text describes the fluorescent markers used in this study to monitor the Start transition: a Whi5-GFP fusion, a *CLN2*pr-Venus-deg transcriptional reporter, and Cdc10-YFP to score budding. (C) The feedback test strains. Strains are isogenic with those described in (B) except that a copy of *GAL1*-*SIC1*-*4A* has been added. Sic1-4A is undegradable, so cells arrest prior to S-phase. The effect of *CLN1,2* feedback on the stability of activation is tested by comparing cells with or without *CLN1,2* (blue text).

The molecular basis of Start has been extensively studied over the last two decades, and many of its key molecular players and their interactions have now been identified (see Figure 1A) [3]. The most upstream regulator of the transition is the G1 cyclin Cln3 [4]. When Cln3 is bound to the cyclin-dependent kinase, Cdk1, the complex phosphorylates the transcriptional repressor Whi5. Whi5 is a repressor of the SBF transcription factor. Phosphorylation inactivates Whi5 and causes its exclusion from the nucleus, leading to SBF-dependent expression of a battery of genes that control early events of the cell cycle [5],[6]. Two additional cyclins, the G1/S cyclins encoded by *CLN1* and *CLN2*, are among the targets of SBF, and are directly involved in bud formation and spindle pole body duplication [3]. They also trigger the degradation of Sic1, a stoichiometric inhibitor of the S-phase cyclins, which allows DNA replication to occur [3]. In addition, high Cln2-Cdk1 activity blocks the mating pheromone pathway by inactivating one of its components, Ste5 [7],[8], and by promoting degradation of Far1, which is itself an inhibitor of Cln2 [8],[9].

Cln1 and Cln2 can promote their own transcription by activating SBF [10],[11], and as has been shown for Cln3-Cdk1, Cln1- and Cln2-Cdk1 can efficiently phosphorylate Whi5 [5],[6]. *cln3* mutant cells are viable, and likely activate SBF via autoactivation of Cln1,2, i.e., through transcriptional positive feedback [12]. However, the functioning of this potential positive feedback loop in wild-type (WT) cells has been less clear; in synchronized cultures, the timing of the appearance of *CLN1,2* mRNAs at the end of G1 is similar in WT and *cln1,2* mutants [13],[14]. Yet, recent work using fluorescent reporters of *CLN2* transcription in time-lapse microscopy assays of single cells demonstrated that the activation of SBF-regulated genes is notably delayed in the absence of Cln1 and Cln2, therefore showing that Cln1 and Cln2 do indeed influence the dynamics of their own activation [15]. The discrepancy between these results is most likely due to the higher resolution of single-cell techniques, which avoid the necessary averaging employed in bulk population studies [15].

Besides modifying the kinetics of gene activation, positive feedback can have diverse consequences on the logic of activation of a gene cascade, depending on the sensitivity and nonlinearity of the autoactivation loop. If the feedback loop is weak, the response of an autoactivating gene to a regulatory stimulus is sigmoidal but continuous and reversible. In the case of strong feedback, the response exhibits discontinuities, jumping sharply from a low to a high state at a high stimulus threshold, and jumping back to the low state at a lower stimulus threshold. Since the low and high thresholds can be significantly different, there is a range of stimuli for which the system has two possible stable states and therefore displays hysteretic behavior. In the case of even stronger feedback, bistability can lead to irreversibility, where the response remains high even when the stimulus is decreased to zero [16].

Since positive feedback does not necessarily make a system bistable or irreversible, it is crucial to record the hysteresis curve of gene activation to characterize its logic, a procedure that has been done in several biological contexts. In the control machinery of the *Xenopus laevis* cell cycle, for instance, bistability in the activation/inactivation of mitotic cyclin-Cdk activity by the Wee1/Cdc25 regulatory circuit has been demonstrated [17]–[19]. The sharp switch in protein kinase activation observed in this bistable system may make mitotic entry irreversible, promoting the unidirectionality of the cell cycle clock.

Similarly, in mammalian cells, the restriction point at the end of G1 has been shown to display bistability in response to growth stimuli [20]. Yet the molecular basis of this behavior could not be unambiguously attributed to the positive feedback of G1/S cyclins. This would require a means of isolating this regulon from the rest of the cell cycle, because other cell cycle regulatory elements, downstream of the G1/S transition, could act to stabilize the high CDK state that is characteristic of the S/G2 phase. Conversely, in budding yeast, inactivation of SBF-mediated expression by mitotic B-type cyclin in yeast [21] precludes observation of the steady-state activity of the G1/S regulon in cycling cells.

To determine whether *CLN1,2*-dependent transcriptional positive feedback in budding yeast Start results in hysteresis or irreversibility, we first examined the sensitivity of the activation of the Whi5/SBF regulatory module in response to cyclins, in the absence of endogenous Cln1,2 feedback. To this end, we modified the wiring of the G1/S regulatory network so that we could precisely trigger its firing using externally controlled reversible pulses of *CLN2* in cells growing in our previously described microfluidics device [22]. Using this system, we showed that Whi5 inactivation and resulting SBF activation exhibit strong nonlinearity, which potentially could make the G1/S transition bistable.

To test this possibility, we examined the long-term stability of activation of the Start regulatory module in the presence or absence of *CLN1,2* transcriptional feedback, following an exogenous pulse of *CLN2*. To achieve this, we blocked B-type cyclin–dependent turnoff of the G1/S regulon by preventing B-type cyclin activation, through controlled expression of an undegradable B-type cyclin inhibitor, Sic1-4A, expressed from the *GAL1* promoter [23]. Using this methodology, we showed that this transition was not only bistable, but also truly irreversible. This irreversibility, which we demonstrated to be due solely to the Cln1/Cln2 feedback loop, provides a solid molecular basis for unidirectional cell cycle “commitment” at Start. A simple mathematical analysis of the system, incorporating parameter values derived from these experiments, confirms that a switch-like behavior of Cln1 and Cln2 expression is expected to occur, which would in turn guarantee a fast and reliable transition from G1 to S phase, despite potentially incoherent *CLN3* input. Thus, these results rigorously dissect the dynamical properties and logic of the Start regulatory module by isolating it from endogenous cell cycle control.

## Results

### Strong Nonlinearity in Start Activation

Since the strength of nonlinearity is critical in establishing the logic of a positive feedback-based regulatory module, we first attempted to quantitatively characterize the sharpness of activation of the *WHI5*/SBF module in response to input cyclin in the absence of *CLN1,2* feedback. Our approach essentially followed the design of previous experiments [24] but looked at single cells.

We used a strain lacking all endogenous G1 cyclins, *Cln1,2,3*, and carrying a copy of *CLN2* under the control of the regulatable *MET3* promoter, see Figure 1B [25]. Previous studies [22] have characterized the following features of this system. First, such *cln1,2,3* mutants undergo a normal G1/S transition when triggered with a 20-min pulse of *MET3pr-CLN2* gene expression (accomplished by exposing the cells to a medium lacking methionine), but are blocked in a pre-Start state when grown in the presence of methionine; second, the cumulative amount of transcription from the *MET3* promoter can be varied by changing the duration τ, of the no-methionine (−Met) pulse; finally, since Cln2p lifetime is about 5–10 min [26], −Met pulses allow for temporally controlled and reversible expression of Cln2p. We therefore used this system to provide varying transient pulses of Cln2, and assayed whether single cells traversed the G1/S transition. *Cdc10-YFP* (a bud neck marker) defined budding and the time of cytokinesis, and we used cytoplasmic relocalization of a *Whi5-GFP* fusion as a reporter for Start activation [5],[12]. Cells were grown in a microfluidic device that allows one to monitor the growth of dividing yeast cells while precisely controlling medium exchanges [22]. Automated time-lapse software was used to record phase contrast and fluorescence images every 3 min over the course of the experiment.

**Figure 2:**
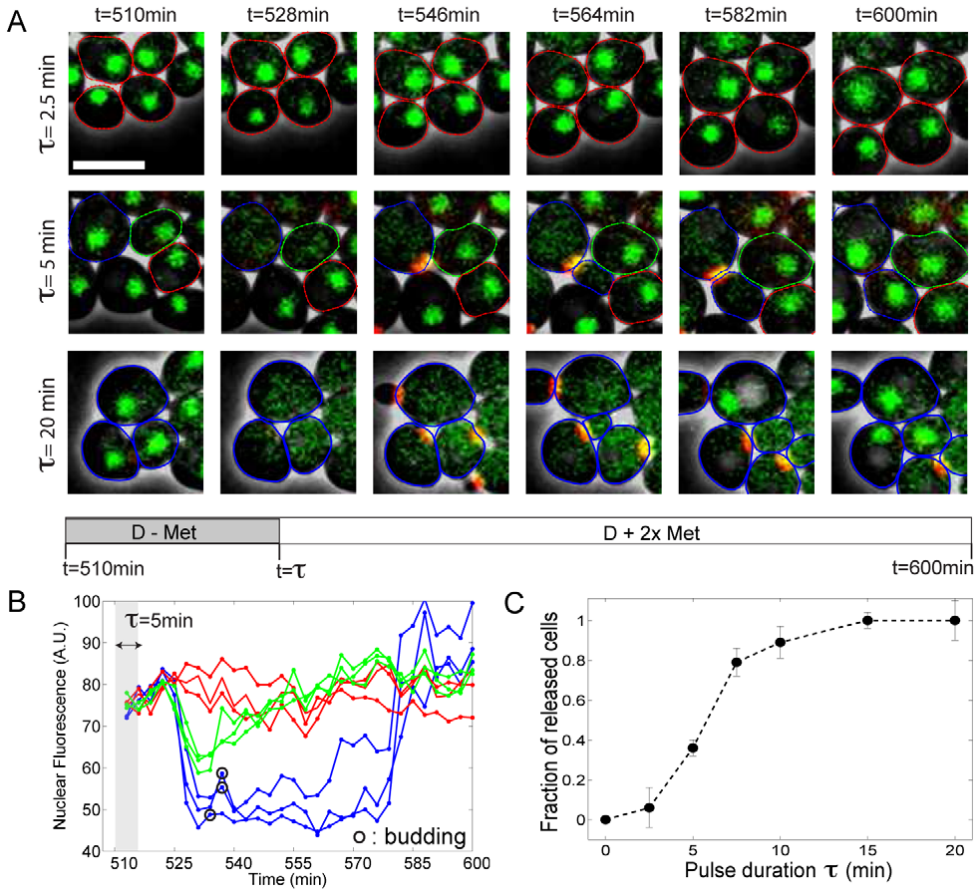
Cells' response to exogenous *CLN2* pulses of various durations. (A) Time series of overlaid images (phase+fluorescence) of *cln1,2,3* cells undergoing Start following a pulse of *MET3pr-CLN2* of duration τ; green signal shows Whi5-GFP, and false-colored red corresponds to Cdc10-YFP. Cell contours have been highlighted to mark different fates of cells: the blue contours mark some cells that undergo Start transition, budding, and subsequent cell cycle completion. The red contour marks a few cells that stay blocked in G1. The green contours mark some cells that undergo partial and reversible WHI5 exit without budding. The white bar represents 5 µm. (B) Quantification of nuclear WHI5-GFP signal (method described in Figure S1) as a function of time for the experiment described in (A). Blue, green, and red lines correspond to released, partially activated, and G1-blocked cells. A.U., arbitrary units. (C) Fraction of cells undergoing Start (released cells) as a function of the pulse duration, τ. Error bars indicate statistical error.

Figure 2A displays sample sequences of overlaid fluorescence and phase contrast images following *CLN2* pulses of varying duration τ. With τ = 20 min (bottom row), all the cells exhibited *WHI5* exit from the nucleus about 18 min after the beginning of the pulse, followed by the appearance of the bud (see red bud-neck marker signal) and subsequent division. In contrast, with τ = 2.5 min (top row), the vast majority of cells stayed in G1 with Whi5 in the nucleus. Pulses of intermediate duration yielded a bimodal behavior; for instance, with τ = 5 min (see middle row and Video S1), about 40% of the cells underwent the Start transition with WT timing and then completed their division normally, whereas 40% stayed blocked in G1 (see blue- and red-marked cells, respectively, in Figure 2A). Stable Whi5 nuclear exit was invariably associated with budding and division, followed by arrest in the next G1 due to *CLN* deprivation.

It was possible that the observed bimodality was due to heterogeneous transcription from the *MET3* promoter across the cell colony, with some cells producing enough Cln2 to pass Start and others producing none. However, with τ = 5 min, we also observed that 20% of the cells underwent a partial and reversible Whi5 nuclear exit, which did not lead to budding (see green-marked cell in Figure 2A and quantification of nuclear fluorescence of cells in Figure 2B; methods used to measure nuclear fluorescence are described in depth in the supplementary Text S1 and Figure S1). These cells clearly imply that there are nonzero levels of Cln2 that are below a threshold for successfully inducing Start.

As we varied the pulse duration, the fraction of released cells increased quite sharply from 0 to 1 (see Figure 2C), suggesting a sensitive response to Cln2 concentration. However, the amount of Cln2 produced does not necessarily scale linearly with the pulse duration. Therefore, we looked for a direct readout of the activity of the *MET3* promoter in each cell. Due to the very short lifetime of Cln2 compared to maturation of fluorescent proteins, we could not detect any fluorescence signal arising from a Cln2-YFP fusion protein. Therefore, we added an independent fluorescent reporter for *MET3* transcription (*MET3pr*-Venus, see Materials and Methods and Figure S2 for the measurement of intrinsic noise).

**Figure 3:**
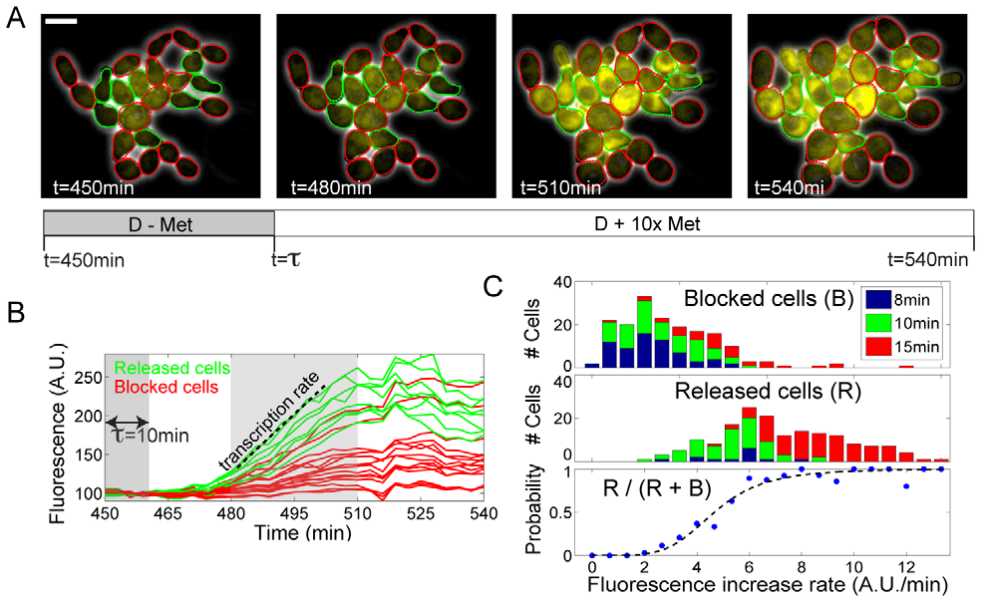
Measurement of the nonlinearity in Start activation. (A) Similar experiments as in Figure 2A, but with the addition of the transcriptional reporter *Met3pr*-Venus and without Whi5-GFP. The images represent overlay of phase and fluorescence at indicated times. Green label corresponds to released cells, and red corresponds to blocked. The white bar represents 5 µm. (B) Quantification of cytoplasmic fluorescence signal as a function of time. Transcription rate is extracted from the rise of fluorescence occurring following the pulse. A.U., arbitrary units. (C) Histogram of transcription rates for blocked cells (top panel) and released cells (middle panel), pooling data obtained with different pulse durations as indicated (total number of data points: 342). The bottom panel shows the probability of budding as a function of the transcription rate, as computed from the two histograms (blue points). The dashed line is the best fit of a Hill function, yielding a Hill coefficient *n* = 4.8±0.3.

With τ = 10 min, we observed the same bimodality in cell fate (see Figure 3A, green cell contours highlight “released” cells undergoing budding, whereas red contours indicate “blocked” cells that failed to bud after the pulse; see also Video S2) that was previously described (see middle panel in Figure 2A). We then used Venus fluorescence from *MET3*-Venus to infer the *MET3-CLN2* transcription rate in each cell. We quantified the distribution of transcription rates, defined as the slope of the fluorescence time traces, for cells that budded after the pulse and cells that failed to bud after the pulse (see Figure 3B; green traces indicate cells that budded; red traces indicate cells that did not bud). We observed that cells that remained blocked had on average lower (but, importantly, nonzero) expression from the *MET3* promoter than released cells, with a fairly tight apparent threshold level of *MET3-CLN2* expression required for subsequent budding. Changing the pulse duration increased the average *MET3* expression (see Figure 3C and Figure S3), but did not modify the observed threshold for budding observed across the population (colored bars in Figure 3C). These experiments thus allowed us to calculate the probability of passing Start as a function of relative transcription rate, over a range of more than one order of magnitude (lower panel in Figure 3C).

There was no correlation between cell size and budding following a pulse, nor between cell size and intensity of cytoplasmic fluorescence, in contrast with previous population measurements (see Figure S4) [24]; this difference may be due to the fact that the cells in this experiment are large due to previous *cln1,2,3* block (average area 1,300 pixels, compared to typical area at budding in WT cells of about 700 pixels [27]; Figure S1B); the size-control effects described by Schneider et al. occurred at considerably lower cell size [24].

The fact that we could observe graded transcription rates in blocked cells (red traces in Figure 3B) demonstrated that these cells likely produced nonzero levels of *CLN2*, therefore excluding the possibility that the observed bimodality was due to on-or-off expression of the *MET3* promoter itself. Fitting the probability of budding to a sigmoid yielded a Hill coefficient of 4.8, indicating a strong nonlinearity in the response of Start to varying Cln2 levels (even in the absence of *CLN1,2* positive feedback). Similar experiments using *WHI5* exit, rather than budding, as a marker for the Start transition gave identical results (unpublished data).

### Investigating the Bistability of Start Activation

Sharp nonlinearity of Whi5 inactivation and subsequent budding in response to Cln2 levels, combined with previous demonstrations of Cln1,2-dependent positive feedback, could yield hysteresis or bistability at Start (see Introduction). In the experiments described in the previous section, the long-term equilibrium stability of the “on” transcriptional state following a *MET3pr-CLN2* pulse could not be addressed, due to the rapid induction of B-type cyclin activity and subsequent repression of SBF [21]. To address this problem, we integrated *GAL1-SIC1-4A*. Sic1 is a potent inhibitor of B-type cyclins [28] that is normally degraded following phosphorylation by Cln1,2. Sic1-4A contains mutated Cdk phosphorylation sites that block its ubiquitin-dependent proteolysis when expressed from the *GAL1* promoter. Sic1-4A thus induces stable post-Start arrest with very low B-type cyclin activity levels [23].

We measured *CLN2* promoter activity with a *CLN2pr-*Venus-degron construct (the degron is the C-terminal PEST sequence of *CLN2*, which destabilizes the fluorescent protein, see Materials and Methods and [12],[22],[29]). We also monitored the localization of a Whi5-GFP fusion as before. Narrow band-pass filters and image processing allowed a quantitatively reliable separation of the Whi5-GFP and *CLN2pr-*Venus-degron signal [22].

In a first set of control experiments, we used a *cln1,2,3 MET3-CLN2 GAL-SIC1-4A* strain with the fluorescent markers described above. Cells were pregrown in the microfluidic device for 9 h, with an appropriate combination of media switches to prepare G1-blocked cells with a large amount of the undegradable SIC1-4A (see Materials and Methods). We then induced *MET3pr-CLN2* for 15 min and observed the following activation of the Start machinery.

**Figure 4:**
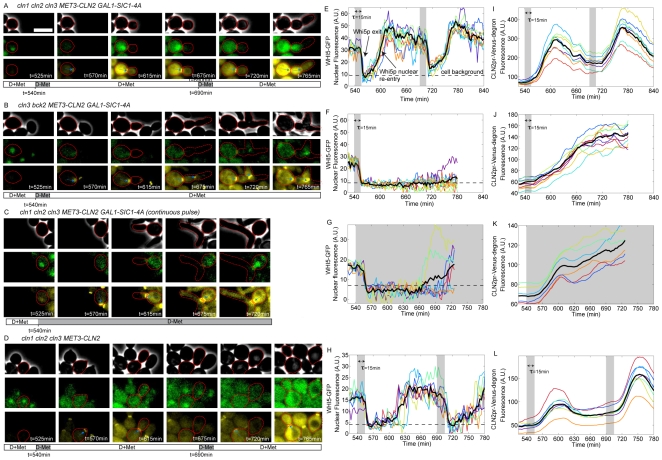
Assay for irreversibility of Start. (A–D) Time series of cells of indicated genotypes undergoing the Start transition following various pulsing protocols (see legend under images for medium switches). The three sets of images correspond to phase, Whi5-GFP signal (green), and CLN2pr-Venus-deg and Cdc10-YFP signals (yellow). Cell contours of interest are marked in red. The bar below the images indicates the timing of medium switches. Blue arrows indicate the bud neck marker Cdc10-YFP; the white rectangle represent 5 µm. (E–H) Whi5-GFP nuclear signal as a function of time. The gray region indicates −Met pulse. Each colored trace represents a different cell. The solid black line is the average over the displayed traces. A.U., arbitrary units. (I–L) CLN2pr-Venus-deg transcriptional reporter signal as a function of time. Each color corresponds to a single cell. The black solid curve is the average over the displayed traces.

The *MET3pr-CLN2* pulse resulted in Whi5-GFP nuclear exit about 20 min later, followed by budding, and a subsequent rise of cytoplasmic fluorescence from the *CLN2pr-*Venus-degron reporter (see Figure 4A, 4E and 4I and Video S3). Interestingly, about 45 min after the beginning of the pulse, we observed Whi5-GFP nuclear reentry and a subsequent decay in Venus fluorescence. These observations suggest that the cells reverted to a “pre-Start” state. Consistent with this idea, a second *MET3pr-CLN2* pulse (150 min after the first) resulted once again in Whi5 nuclear exit, *CLN2pr-Venus-deg* expression, and rebudding of the already budded cells. The 45-min delay in reaccumulation of Whi5 in the nucleus should be compared to the ∼10-min half-life of the Cln2 protein, implying some slow step in reversal to a pre-Start state; this time delay could reflect the time required for Whi5 dephosphorylation, but we lack direct evidence for this.

These results suggest that, in the absence of endogenous cyclins, the Start transition is fully reversible, and that SBF-regulated genes are transcribed only transiently following activation by a transient exogenous Cln2 pulse, after which Whi5 reenters the nucleus and repression is reestablished.

We then carried out the identical protocol, but in cells containing functional *CLN1,2*. In addition, we introduced a mutation in *BCK2* in these cells. Bck2 allows *CLN1,2 cln3* cells to fire *CLN1,2* expression spontaneously, in the absence of either *CLN3* or exogenous *MET3-CLN2* expression [30],[31], and it was important for our experimental design to maintain strict exogenous control of *CLN1,2* expression by *MET3-CLN2*. Identical results to those described above were obtained in *bck2 cln1,2,3* cells (unpublished data). In contrast, strikingly different results were obtained in *bck2 CLN1,2 cln3* cells: following the pulse, Whi5 exited the nucleus and remained in the cytoplasm, and *CLN2pr-*Venus-degron expression was activated and remained on for at least 3 h after the transient exogenous *MET3pr-CLN2* pulse (compare Figure 4B, 4F and 4J to Figure 4A, 4E and 4I; see also Video S4). This was true in 12/15 cells examined; in three cells, Whi5 reentered the nucleus several hours after the pulse, but even in these cells, reentry was strikingly slower than in the *cln1,2* background. Thus, *CLN1,2* could apparently maintain their own expression once activated. This was observed in the absence of Bck2, but it is likely that they could also do so in the presence of Bck2, since Bck2 is a Cln-independent activator of *CLN1,2* expression. Consistent with these results, WT (*CLN1,2 BCK2+*) cells blocked at the G1/S border using a pulse of GAL1-SIC1-4A exhibited no turnoff of the *CLN2* promoter (unpublished data), also controlling for unexpected effects of *BCK2* deletion.

Thus, we infer from these results that following the initial exogenous *MET3-CLN2* pulse, endogenous *CLN1,2* were activated, and thenceforth maintained their own expression by positive feedback. As an alternative means of assessing the phenotype of continuous *CLN2* expression, we tested a *cln1,2,3* strain with continuous expression of *MET3-CLN2* in the presence of stable Sic1-4A. The results were similar to those obtained with only a transient pulse of *MET3-CLN2* in a *CLN1,2* background: a very long period with Whi5 out of the nucleus, and continuous *CLN2pr-*Venus-degron expression (Figure 4C, 4G, and 4K).

As a final control, we tested transient induction of *MET3-CLN2* in a *cln1,2,3* strain, without first inducing *GAL-SIC1-4A*. Following the pulse of exogenous Cln2, these cells underwent a normal, complete cell division cycle (Figure 4D, 4H, and 4L). Notably, Whi5-GFP nuclear reentry was observed about 60–80 min after the beginning of the pulse (Figure 4H), as opposed to about 45 min when B-type cyclin activation was blocked by Sic1-4A (Figure 4E). This is presumably due to Whi5 phosphorylation by Clb-Cdk even after Cln1,2 disappearance [5], indicating a critical distinction in WT cells between initiation and maintenance of the post-Start state.

Thus, following transient activation, continued expression of SBF-regulated genes requires transcriptional positive feedback through expression of *CLN1,2*. Remarkably, the presence of this feedback ensures the maintenance of the G1/S program over a timescale well beyond the duration of the cell cycle. Therefore, the Start regulatory module behaves as a ratchet that ensures the irreversibility of this cell cycle transition.

### Whi5 Imposes the Requirement for Cln1,2-Dependent Positive Feedback for Start Irreversibility

**Figure 5:**
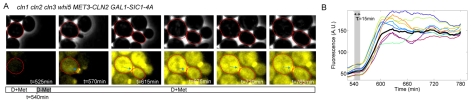
Influence of Whi5 on Start irreversibility. (A) Time series of images (phase, Cdc10-YFP, and CLN2pr-Venus-deg fluorescence signals) of *cln123 whi5* delta cells following a 15-min *MET3pr-CLN2* pulse. The red contour shows a typical cell of interest. (B) CLN2pr-Venus-deg fluorescence signals observed in (A). Each color corresponds to a different cell. The black solid line is an average of the displayed cells. A.U., arbitrary units.

In the absence of *CLN1,2*, a pulse of exogenous *MET3pr-CLN2* produced only a transient activation of SBF-dependent gene expression. We hypothesized that this could be specifically due to reentry of the Whi5 repressor into the nucleus. To test this idea, we pulsed a *cln1,2,3 whi5* strain with *MET3pr-CLN2* in the presence of Sic1-4A (with the same protocol as in Figure 4B, which demonstrated Start reversibility in the absence of *CLN1,2* feedback). In this background, following the exogenous *CLN2* pulse, the transcriptional reporter *CLN2pr-*Venus-degron stayed “on” throughout the experiment (Figure 5A and 5B).

This supports the role of Whi5 as a powerful repressor of SBF-driven genes, which can only be countered by *CLN1,2* feedback; in its absence, SBF, once activated, will continue to promote transcription of its target genes indefinitely (compare Figure 5A and 5B to Figure 4A and 4E). We assume that the constant plateau level of Venus-degron achieved in these cells represents a balance between continued synthesis and degradation, since the Venus-degron degradation rate was shown to be invariant throughout the cell cycle [22]. The rapid loss of Venus-degron signal in the parallel experiment in a *WHI5* background, where transcription is presumably turned off, provides a control indicating continued transcription in the absence of Whi5.

### CLN2 Control of Budding Dynamics

**Figure 6:**
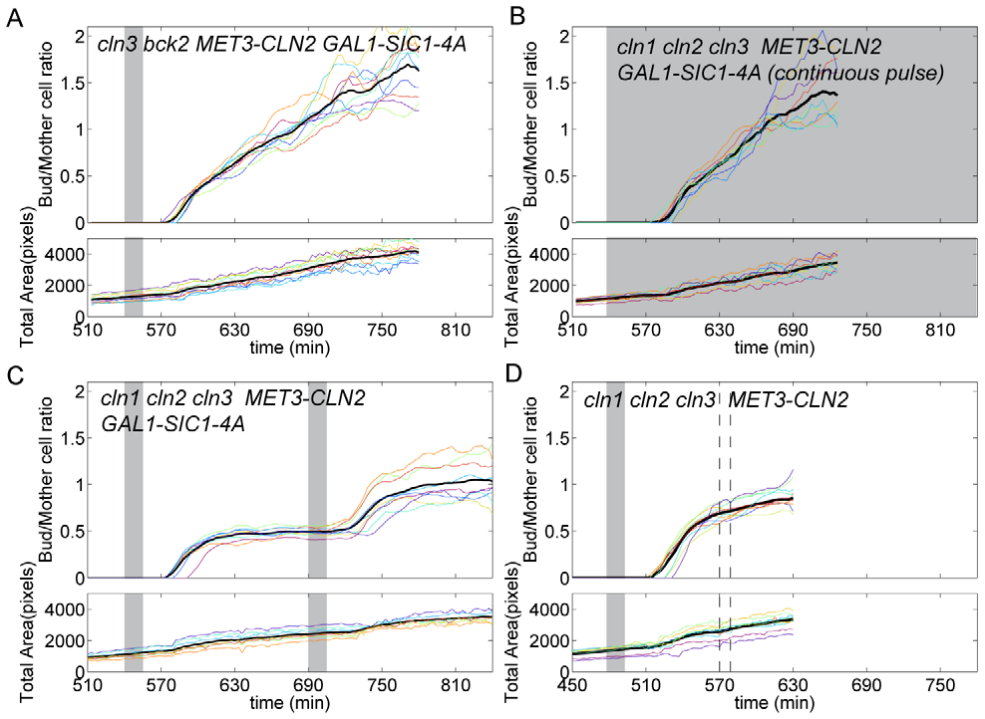
Polarized growth with or without feedback. (A–D) Bud to mother size ratio (top panel) and total (mother+bud) size of cells as a function of time for cells undergoing Start activation. Each plot corresponds to a different strain background, as indicated. Each color represents a different cell, and the solid black line is an average over the displayed traces. Shaded areas indicate −Met pulses. In (D), the interval between the dashed line roughly indicates the time by which nearly all cells have completed division.

To further characterize the influence of *CLN1,2* feedback on the dynamics of the cell cycle, we also monitored the dynamics of bud growth following a pulse of exogenous Cln2, in Sic1-4A-blocked cells (*cln1,2,3* or *CLN1,2 cln3*). We obtained a quantitative measurement of polarized growth (independent of overall cell growth) by calculating the ratio of the bud size to its mother's size as a function of time (Figure 6).

In our system, following induction of positive feedback (*CLN1 CLN2 cln3 bck2*, following Sic1-4A expression and a brief *MET3-CLN2* pulse), we observed that most or all new cell mass (up to a tripling of initial cell size) was transferred to the bud(s), implying a strongly polarized growth pattern (see Figure 4B and Figure 6A for the quantification of that effect). This is consistent with continuous cytoplasmic Cln2 activity, as Cln2 is known to be directly involved in both bud formation and elongation. To further support this interpretation, a continuous exogenous *CLN2* pulse in a *cln1 cln2 cln3* background yielded the same polarized growth pattern (see Figure 4C and Figure 6B).

In striking contrast, a transient *CLN2* pulse done in the absence of *CLN1,2* allowed polarized bud growth for only approximately 45 min, at which time the bud–mother ratio saturated at a value close to 0.5 (Figure 6C; overall growth of the mother+bud system continued to increase over the course of the experiment, see lower panel). Interestingly, the bud–mother size ratio obtained at saturation in this experiment was close to (though slightly smaller than) the known ratio obtained at division in WT cells, which is about 0.65 [27] (see also control experiments without *GAL1-SIC1-4A* expression in Figure 6D). These results are consistent with the observations of McCusker et al. [32], who showed that Cdk1 activity was continuously required for polarized bud growth; our results show that Cln1,2 are the most likely activators of Cdk1 for this activity. (McCusker et al. also found that overall cell size did not continue to increase in the absence of Cdk1, in contrast to our results.)

### A Simple Ordinary Differential Equation (ODE) Model for Start Activation

How could *CLN1,2* positive feedback make Start entry an irreversible switch? To understand the emergence of this property, we turned to a mathematical description of the Start regulatory module. One strategy to model biological gene networks is to describe each component at the molecular level using ordinary differential equations (ODEs). A drawback of this approach is that it uses many experimentally unknown variables and parameters. In addition, often the complexity of ODE models obscures the governing mechanisms. Therefore, we chose to look for a simple model that would rely on parameters whose values could be estimated from our experiments.

Our model attempted to calculate, as a function of a given G1 cyclin input concentration (Cln3 or exogenous Cln2), the steady-state concentration of endogenous Cln1,2 protein (we name the two homologous proteins *Cln2* for simplicity), which are degraded at a rate δ, and whose production is controlled by all G1 cyclins present in the cell. We assumed that G1 cyclins (*Cln2*, *Cln3* or exogenous Cln2 (*Cln2e*)) promote *Cln2* production in a nonlinear manner following a sigmoidal law, parameterized by α, *n*, and *K* (see Equation 1) :
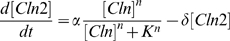
(1)with [*Cln*] = [*Cln2*]+[*Cln3*]+[*Cln2e*]; α is the maximum production rate, *n* is the Hill coefficient that characterizes the nonlinearity in *Cln2* production as a function of input cyclins, and K is the cyclin concentration at which *Cln2* production has reached its half-maximum. The experiments described in Figure 3 have revealed that the probability of Start transition is a sharp sigmoidal function of exogenous *CLN2* driven by the *MET3*pr (in the absence of endogenous Cln1,2 feedback). Figure 2 also demonstrated that Whi5 nuclear exit and budding are perfectly correlated markers of the Start transition, as no budding is observed if Whi5 does not stably transit to the cytoplasm. Therefore, since the expression of endogenous Cln1,2 is directly controlled by the Whi5/SBF module, we hypothesized that the cyclin-dependent *Cln2* production rate would exhibit the same nonlinearity observed experimentally, i.e, *n*∼4.8.

In this formulation, we did not describe the dynamics associated with other chemical reactions such as protein binding, phosphorylation, transcriptional events, or mRNA degradation. These events likely occur on a fast timescale compared to the main dynamics of the system (Cln2 protein lifetime is about 5–10 min); therefore, these reactions do not need to be modeled explicitly.

These simplifications reduced the system to a single variable problem (endogenous *Cln2*), with the two internal parameters *n* and K. The system was rendered dimensionless by 1) normalizing time by 1/δ (scaling to *Cln2* lifetime), and 2) scaling concentration to the ratio of α/δ; the corresponding *Cln2* concentration was renamed *X*, so that Equation 1 becomes :
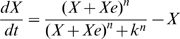
(2)with k = Kδ/α, X = [*Cln2*]δ/α, and Xe = ([*Cln3*]+[*Cln2e*])δ/α.

We then wanted to determine the dependency of the steady-state value of *X* on a given input *Xe* (either the natural endogenous inducer *Cln3*, or a controlled pulse of exogenous *Cln2*), and how this dependency changes as *k* and *n* vary.

**Figure 7:**
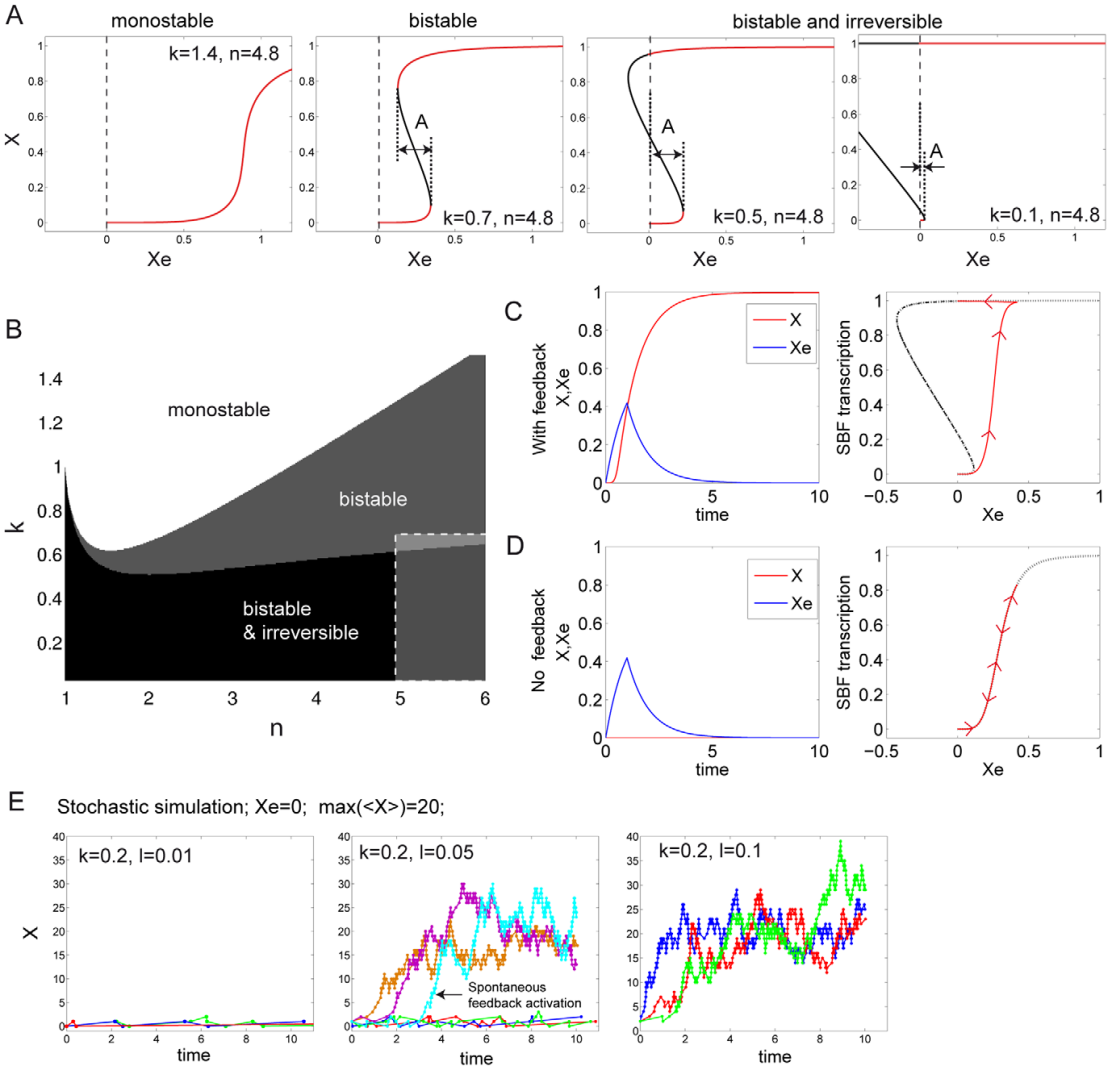
Model of Start activation. (A) Steady-state value of *X* as a function of input cyclin, *Xe*, for different values of (*n*, *k*) as indicated on the plot, revealing different classes of behaviors: monostability, bistability, and irreversibility. The red line segments indicate possible stable states, whereas black line segments show unstable regions. The amplitude, A, of the bistability region is indicated. (B) Map of the different possible behaviors as a function of the parameters *n*, *k*. The dashed region indicates the subsection of the map where the experimental system is thought to function. (Please note that the *n* = 1 or *k* = 0 cases yield limit monostable behaviors.) (C) Left panel: simulation of the temporal response of *X* as a function of a transient pulse of *Xe* in the presence of positive feedback. Right panel: SBF transcription (assumed to be proportional to the first term in the right-hand side of Equation 2) as a function of *Xe*. Arrows indicate the direction of the trajectory. (D) Same as [C]), except that the positive feedback is removed (*X* = 0). (E) Stochastic simulation (following Gillespie's method [44]) of *X* activation in the absence of any input (*Xe* = 0) but after adding a basal transcription term l (0<l<1) to the right-hand term of Equation 2. The value of *n* used was 4.8, the values of other parameters are indicated. To control the amplitude of statistical fluctuations in protein number, the value of alpha was adjusted such that the maximum number of proteins is on average 20. Left, middle, and right panels show sample temporal traces for different values of l. Each trace represents a single cell.

Choosing *n* = 4.8, Figure 7A shows the behavior of the system for different values of *k*, obtained by numerically solving Equation 2. With *k* = 1.4, plotting *X* as a function of *Xe* shows a sharp dependency of *X* upon *Xe* when *Xe*∼0.9. However, *X* takes on continuous values between 0 and 1 as *Xe* goes from 0 to values larger than 1, see left plot in Figure 7A. The system is monostable, since one value of *Xe* corresponds to a single value of *X*. With *k* = 0.7, we observe a bifurcation into a bistable system: there is an interval of input concentration *Xe* for which *X* can take two different values, characterizing states of low and high transcription (see red curve segments), with no possibility of observing an intermediate state (the black curve segment describes unstable states). The switches between these two different stable states can occur freely as one varies *Xe*, yet the threshold *Xe* values at which the system switches up and down are different, so that the system displays hysteresis. When *k* further decreases, as the black segment crosses the ordinate axis on the *X* versus *Xe* plot, the possibility of switching from the high state to the low state by decreasing *Xe* disappears (or, literally, occurs at negative *Xe*, which is biochemically impossible); see the two right-hand plots in Figure 7A. The only possible transition is from the low state to the high state. The system is therefore irreversible.

Figure 7B provides a classification of the three possible behaviors described above in the (*n*, *k*) space. The partitioning between monostable, bistable, and irreversible domains was deduced analytically from Equation 2 (see Text S1 for details). This map shows that the behavior of this system depends strongly upon the assigned values of parameters. Where does the experimental system fit in this map? We showed (see Figure 3) that the nonlinearity in the activation of the Whi5/SBF module could be characterized by a Hill coefficient of *n* = 4.8 or higher. From our previous studies [22], we knew that the transcription rate from the *MET3* promoter is about 0.7 times that of the maximally induced *CLN2* promoter. Therefore, no matter the duration of the pulse, the exogenous concentration of *CLN2* from the *MET3*pr [*Cln2e*] is such that [*Cln2e*]<0.7 α/δ (α/δ is the maximum concentration of Cln2, when expressed from its endogenous promoter). Since we observed reliable induction of Start using short pulses of *CLN2* driven by the *MET3* promoter, we assumed that the exogenous Cln2 produced was high enough to trigger substantial transcription of SBF driven genes, i.e., [*Cln2e*]*^n^*/[*Cln2e*]*^n^*+K*^n^* is close to 1. This implies that [*Cln2e*]>K, and consequently, *k*<0.7 (and probably significantly smaller). According to this numerical analysis, the experimental system is thus expected to work in an irreversible manner (see the region delimited by the white dashed line in Figure 6C), as was indeed observed experimentally.

To illustrate this functional mode, we integrated Equation 2 over time, assuming that the input was a transient pulse of exogenous *Xe* (following first-order degradation kinetics, as *X* does, and being produced at a rate of 0.7 over two units of time, i.e., 10–20 min), using the parameter values *n* = 4.8 and *k* = 0.2. In the presence of *X* (i.e., with feedback), the pulse of *Xe* triggered activation of the feedback loop so that *X* converged to ∼1 (left panel in Figure 7C). Plotting SBF transcription (as defined by the first right-hand term in Equation 2) as a function of *Xe* best illustrates the effect of this irreversibility, as SBF never turns back off after *Xe* concentration has decreased to 0. In contrast, in the absence of endogenous cyclin (*X* = 0, i.e., no feedback), the activation of SBF is transient, as illustrated by the red arrows on the curve in the right panel of Figure 7D.

Stability plots obtained with *k* = 0.5 and *k* = 0.1 (decreasing *k* results in increasingly efficient activation of the CLN2 promoter by Cln protein) had quite different ranges of bistability (Figure 7A, Figure S5A). Decreasing *k* lowers the critical threshold of *Xe* required to achieve a stably activated transcription state. Thus, with small enough *k*, the feedback loop could autoactivate, provided that the transcriptional leakiness of *X* and/or the fluctuations in the number of molecules of *X* were high enough.

To test this possibility, we ran a stochastic simulation of a modified version of Equation 2 that includes a transcriptional leakiness variable l (0<l<1) and takes the number of molecules into account (see Text S1). Assuming a mean number of molecules <*X*> = 20 in the activated state (the number of actual Cln2 proteins is known to be much higher, but the number of *CLN2* mRNAs is probably in this range, which sets the amplitude of statistical fluctuations), we indeed observed frequent autoactivation of the feedback loop in the complete absence of input (*Xe* = 0) with l values as low as 0.05 (i.e., when basal transcription represents 5% of the maximum transcriptional level), see Figure 7E. Changing the average number of molecules involved did not qualitatively change this result, as shown by plotting the probability of observing auto-activation (within 10 units of time) as a function of l and <*X*> (Figure S5B). The frequency of autoactivation appeared to be mainly dependent on the relative values of *k* and *l*, as intuitively expected (Figure S5C). It was beyond the scope of this study to obtain accurate estimates of *k* and *l* and therefore determine in which subregion of the (*k*, *l*) map the experimental system belongs. However, as *cln3* mutants (i.e., no *Xe* input) are not blocked in G1, but *cln3 bck2* mutants are, *BCK2* deletion most likely reduces leakiness in basal (Cln-independent) *CLN2* transcription [30],[31]. Thus, viability of *cln3* and inviability of *cln3 bck2* cells could be accommodated in our framework by a lowering of l due to *bck2* deletion.

## Discussion

In this study, we report the combined use of yeast molecular genetics and biophysical methods to study the logic of the Start regulatory module. A logical way to get an understanding of the functioning of a complex biochemical network is to decipher the individual properties of its component pathways. To this end, we isolated the Start pathway from the rest of the cell cycle network by partially rewiring its architecture and connections. Using a microfluidics device, we could observe single cells over the course of our experiments, and control the cell environment in order to trigger reversible pulses of gene expression. This allowed us to reliably investigate the steady-state properties of the Start regulatory module despite the highly dynamic nature of the cell cycle.

*CLN1,2*-dependent positive feedback was shown to promote coherent expression of the SBF/MBF regulon [15]. Here, we demonstrate another role of this positive feedback: irreversibility of the Start transition. We show experimentally and by empirically constrained modeling that this irreversibility is quantitatively consistent with the strength and nonlinearity of the transcriptional response to Cln protein levels. We show further that dependence of irreversibility on the positive feedback loop is enforced by the Whi5 repressor. Even in the absence of Whi5, the transcriptional system is still off in a *cln1,2,3* background until the exogenous *CLN2* pulse, indicating that there must be another step in Cln-dependent activation of transcription besides Whi5 inactivation, as concluded previously [5],[6],[15]. Nevertheless, the dominant logic that emerges from this regulatory module is control by counteracting forces—*CLN1,2* positive feedback versus Whi5-mediated repression. Active counterbalancing forces are frequent and possibly necessary functions in ultrasensitive, hysteretic, or irreversible systems. Our results suggest that this antagonism may dominate Start dynamics, even though other mechanisms exist.

Our system necessarily required forceful modification of the WT genotype, to isolate the circuits under study. Nevertheless, the properties we observe are likely to be relevant to Start in WT cycling cells. First, the timing is compatible with cell cycle timescales; our model suggests that, in the presence of feedback, the time it takes to turn on SBF-regulated genes is set by the Cln1,2 degradation time, which is on the order of 5–10 min. Since *CLN2* is known to be “on” for 20–30 min during a normal cell cycle, it is very likely that the switching between the two described stable states can occur within that time window. Additionally, the relatively short time it takes for Whi5 to relocate to the cytoplasm (about 6 min [12]) supports the idea that the module reaches steady state in any given cell cycle, even though it is subsequently efficiently inactivated by B-type cyclin activity.

Irreversibility of Start has the potential to make the system very reliable in terms of information processing of pre-Start signals, no matter how noisy and/or persistent these inputs are. Although some of the timing variability in Start is due to cell-size control, a substantial residual amount of variability is likely due to gene expression noise [33]. Cln3 protein, which is the most upstream regulator of Start, is present in low abundance and is highly unstable [34]–[36]. Furthermore, its variations during the cell cycle are quite modest in comparison to other cyclins [4],[37],[38]. In this context, an interesting feature of such a highly excitable irreversible switch is to allow a short-lived Cln3 fluctuation above the transition threshold to trigger the robust firing of Start (provided the duration of this fluctuation is equal or larger than the typical duration of activation of the Cln1,2 feedback, which is set by Cln2 halftime, as mentioned above).

This function stands in striking contrast with some signal transduction pathways in which the intensity of the response depends on the amplitude of the input. Here, the architecture of the system implies that the entry into a new cell cycle is an all-or-none decision, which cannot be reversed. Unidirectionality of cell cycle transitions has been attributed to proteolysis of key regulatory proteins [39],[40]. In contrast, several recent studies stress the functional importance of bistable regulatory modules that induce “systems-level” irreversibility [16],[41]. Our study of the G1/S transition suggests that these two mechanisms are likely to be intertwined, as the self-sustained activation of *CLN2* ensures complete degradation of the B-type cyclin inhibitor Sic1, a key step in activation of B-type cyclin activity and irreversible progression into S phase and mitosis, even after Cln-dependent transcription is shut off.

Other signals, including those from the mating pathway, are integrated by the Start regulatory module. In the presence of pheromone, pre-Start cells block in G1 with inhibited Cln proteins and very low *CLN2* transcription, whereas post-Start cells are insensitive to pheromone due to Cln-dependent degradation of the Far1 inhibitor and inactivation of the Ste5 signal transduction component [7]–[9]. The sharp transition that arises from the positive feedback-mediated switch may similarly sharpen the transition between the pheromone-sensitive and pheromone-resistant phase, avoiding intermediate, potentially deleterious responses, such as having a budded cell with replicated DNA undergoing mating arrest. Knowing that the Cln2 degradation rate sets the timing for feedback activation, it is tempting to speculate that this rate has been evolutionarily tuned to speed up the transition and therefore to prevent contradictory signals and “stuttering” at Start.

Modeling the effect of Cln1,2 positive feedback using a single-variable equation allowed us to test the qualitative validity of our interpretation of our experiments. One goal of the theory of dynamical systems is to classify complex systems according to their steady-state behavior, assigning them to simple categories such as bistability, irreversibility, etc. A mathematical description of a dynamical system is then relevant when it lets one measure the importance of each parameter on global dynamics. To do so, the model must rely on a limited number of those key parameters, each of whose values can be extracted experimentally. We found the qualitative behavior of the Start transition to be very dependent upon two parameters, *k* and *n*, which together characterize the strength of SBF activation as a function of inputs. Using our experimental estimates of *n* and *k*, our model predicted bistable and irreversible behavior.

Nonlinearities in the Cln-dependent activation of SBF and Cln1,2-dependent positive feedback are the key aspects governing the logic of the Start transition. Further biochemical studies will be necessary to determine the molecular origins of these nonlinearities. Cooperativity in the phosphorylation of multiple Cdk sites on Whi5, for instance, could generate an ultrasensitive response, as already observed for other Cdk targets [42]. Consistent with this idea, reducing the number of Whi5 phosphorylation sites decreases coherence of expression of the SBF/MBF regulon [15].

In addition, the model revealed that, in this functional regime, the stability of the nonactivated state sharply decreases as the efficiency of Cln-dependent activation of *CLN2* expression increases (decreasing *k* in our model). In fact, we found numerically that the feedback loop could be triggered in the presence of some *CLN2* transcriptional leakage without any external input. This scenario, which appeared to be plausible with reasonable parameter values, provides a potential explanation for the role of Bck2 in tuning the stability of the unactivated state; we speculate that Bck2 could, by raising *CLN2* transcriptional leakage, favor a stochastic transition to a post-Start state, which would explain why cln3 mutant cells do not arrest in G1, but exhibit high variability in G1 time [12]. Thus, our results are not only relevant to the overall dynamics of the Start module, but can also provide insight into the effects of noise in gene expression at Start [12],[33].

## Material and Methods

### Strains and Plasmids

All strains were constructed either by transformation (see below) or by mating and tetrad analysis, using our standard lab stocks (all W303 background) as starting material (see Table S1 for list of strains and plasmids). *MET3pr*-Venus, *MET3pr*-Venus-degron, and *MET3pr-CLN2* constructs were integrated at the *URA3* locus by StuI digestion of pCL25, pCL10, and pCL17, respectively. *MET3pr*-Venus-degron was integrated at the *TRP1* locus by XbaI digest of pGC25D. *MET3pr-*mCherry and *MET3pr*-Venus were integrated at the *MET3* locus by MfeI digest of pCL13 and BsmI digest of pGC25, respectively.

### Growth Conditions and Media

Before starting time-lapse experiments, cells were pregrown overnight in standard synthetic complete medium supplemented with dextrose (SCD), raffinose (SCR) (with or without galactose [G]), and with the appropriate Met dosage. The standard 1× Met concentration was set to 0.02 g/l. We used 2× Met to repress the expression of *MET3pr-CLN2*, except for the experiments described in Figure 3, in which 10× Met was used to obtain a lower fluorescence level from *MET3pr*-Venus in the repressed state. The temperature was set to 32°C in the microfluidic setup, which ensured a growth rate very close to the optimal one defined in liquid cultures.

### Exogenous Cln2 Expression from the *MET3pr*

In experiments described in Figures 2 and 3, we induced Cln2 expression from the *MET3pr* promoter by doing short and reversible pulses of medium lacking methionine, as previously described [22].

In Figure 3, we added an independent fluorescent reporter for *MET3* transcription (*MET3pr*-Venus). We showed that two *MET3pr*-fluorescent protein fusions in the same cell showed highly correlated expression of the two fluorescent proteins: the intrinsic (intergene) noise is 0.17, whereas extrinsic (correlated) noise is 0.37 (computed according to [43]; Figure S2), implying that detection of YFP in *MET3pr-CLN2 MET3pr-YFP* cells implied simultaneous expression of *MET3pr-CLN2* in >80% of the cells. In this strain, we also integrated *GAL1pr-CLN1* in order to allow pregrowth of the cells in medium containing saturating methionine (synthetic complete+raffinose+1% galactose+10× Met, SCRG+10× Met) so that very little cytoplasmic fluorescence could accumulate during the period preceding the *CLN2* pulse. This approach allowed us to measure increases in Venus fluorescence levels even in response to very short −Met pulses, with a high signal-to-noise ratio. Cells continuously expressing *CLN1* from the *GAL1* promoter during pregrowth looked elongated, as a result of an artificially high G1 cyclin concentration; see Figure 3A.

### Destabilized Transcriptional Reporter (Venus-Degron)

The Venus-degron fusion (the degron is the C-terminal PEST sequence of *CLN2*) has proven to be a reliable reporter to monitor the transient activation of transcription of a given promoter [22]. Although stability of the Cln2 protein itself is controlled by Cdk phosphorylation [26], and therefore might be cell cycle regulated, our previous data show no cell cycle regulation of Venus-degron stability [22]. This is important for the present study since changes in reporter level imply changes in production rate

### Preparation of Cells for the Testing of Start Stability

Cells were pregrown in the microfluidic device for 5.5 h in SCR medium lacking methionine (SCR-Met; *MET3pr-CLN2* on, *GAL-SIC1-4A* off), which allowed for approximately three divisions. They were then grown in SCR+Met (*MET3pr-CLN2* off, *GAL-SIC1-4A* off) for 1.5 h to block the cells in G1 due to *CLN* deprivation. We then flowed SCRG+Met medium (*MET3pr-CLN2* off, *GAL-SIC1-4A* on) for 1.5 h to allow cells to accumulate SIC1-4A. The medium was then switched to SC glucose+Met (SCD+Met; *MET3pr-CLN2* off, *GAL-SIC1-4A* off) for half an hour to acclimate cells to glucose medium. Finally, cells were pulsed for 15 min with SCD-Met (*MET3pr-CLN2* transiently expressed, *GAL-SIC1-4A* off). We used glucose in the latter steps of the experiment because cells grew better in glucose than in raffinose or galactose, and sufficient stable Sic1-4A had accumulated during the galactose pulse to effectively block B-type cyclins for the remainder of the experiment.

### Microscopy

Images were acquired using a motorized Leica DMI6000B microscope with a 63× N.A. 1.4 objective and a Hamamatsu Orca-AG camera. Fluorescence illumination of the samples was achieved using a standard mercury lamp and high-speed Uniblitz shutters. Image acquisition was driven by custom Matlab software, as previously described [22]. Images (phase contrast + fluorescence) were acquired every 3 min. Up to eight fields of view could be monitored with this interval timing.

### Microfluidic Device

We used the microfluidic setup reported in Charvin et al. [22]. All the methods and protocols regarding the handling and the preparation of the sample are described there in detail. We used an array of four three-way electrovalves to control medium switches in a programmable manner (using Matlab).

### Data Analysis

Phase contrast images of growing cells were segmented using custom Matlab software, as previously described [22]. Mean cytoplasmic fluorescence was measured by averaging pixel intensities within a cell contour. Whi5-GFP nuclear fluorescence was scored using a custom Monte Carlo procedure described in the Text S1.

### Mathematical Model

The steady-state properties of the system described by Equation 2 were derived analytically; see Text S1 for details. Further properties of the model, such as the amplitude of bistability, were calculated numerically. Integration of the deterministic model described by Equation 2 was done using a custom Matlab program. Stochastic simulations were done using Gillespie's algorithm [44]. All model parameters and values are defined in the text and figures.

## Supporting Information

Figure S1 **Detection of nuclear fluorescence.** (A) Fluorescence images showing the Whi5-GFP in WT cycling cells at indicated timings. The dark blue line indicates the cell contour (retrieved from phase contrast images). The cyan circle shows the position of the nucleus as determined by scoring Whi5-GFP using a custom procedure shown in Text S1. The white rectangle represents 2.5 µm. (B) Same data as in (A), but also displaying the permanent nuclear marker Htb2-mCherry (top images). This marker was used to retrieve the actual position of the nucleus (cyan line), which in turn allowed quantification of the Whi5-GFP nuclear signal (bottom images). (C) Quantification of nuclear signal according to methods described in (A) (blue lines) and (B) (red lines), as a function of time. Solid lines show nuclear signal, whereas dashed line represent cytoplasmic signal. Black rectangles indicate data points shown in (A) and (B).
(0.39 MB TIF)

Figure S2 **Correlation of expression of MET3-driven fluorophores Venus and Cherry.** (A) Top panel: time series of images (overlay of phase plus fluorescence) of a cell colony following a 20-min pulse of −Met. Cells carry MET3-Venus (false colored in green) and MET3-mCherry (colored in red). Bottom panel: phase images as in top panel, plus contours of scored cells (blue lines). White lines indicate cell parentage. (B) MET3-Venus fluorescence trace as a function of time, corresponding to the experiment described in (A). Each colored curve corresponds to a single cell. The shaded area represents the −Met pulse. (C) Same as (B), but for MET3-mCherry. The color coding is consistent with (B). mCherry has a longer maturation half-time than Venus (resp. ∼45 min, unpublished data, versus 18 min, see [22]), thus explaining the observed delay in the rise of fluorescence in (C), as compared to (B). (D) Correlation of transcription rate (as defined by the fluorescence increase rate in [B] and [C]) in the linear part of the curves, and normalized to the mean of each distribution) of the two markers Venus and mCherry over a population of cells. Each blue point corresponds to a single cell. The solid black line is the diagonal.
(0.87 MB TIF)

Figure S3 **Cell–cell average transcription rate from the MET3pr (MET3pr-Venus construct) as a function of pulse duration (calculated as reported in** **Figure 3** **).** Error bars indicate standard deviation. Each data point shows an average of around 100 cells.
(0.06 MB TIF)

Figure S4 **Dependence of Start activation and MET3pr transcription on cell size.** (A) Top panel: histogram of cell area (pixels) at division for blocked and released cells, as described in Figure 2F. Bottom panel: probability of passing through Start as a function of cell size. (B) Correlation plot between MET3pr transcription rate and cell area. The coefficient of correlation (corr) is indicated.
(0.17 MB TIF)

Figure S5 **Amplitude of bistability and probability of Start autoactivation as a function of model parameters.** (A) Amplitude A of the bistability region as a function of *n* and *k*, calculated numerically using the deterministic model described by Equation 2. (B) Probability of observing feedback autoactivation (within 10 units of time) as a function of l and average protein number <*X*>, using a stochastic version of the model (see Text S1 for details). (C) Probability of feedback autoactivation as a function of *k* and the leakiness *l*, using a stochastic simulation of the model.
(0.26 MB TIF)

Table S1 **List of strains and plasmids.**
(0.02 MB DOC)

Text S1 **Model details and complementary methods.**
(0.08 MB PDF)

Video S1 **Start activation of** ***cln1 cln2 cln3*** **cells in response to a 5-min-long pulse of exogenous** ***CLN2*** **(corresponding to the data described in** **Figure 2** **).** The left panel shows phase contrast images. Cell contours are highlighted using different colors, depending on the response of each to the pulse: released cells (blue), G1 blocked cells (red), transiently activated cells (green), or dead cells (white); the right panel shows fluorescence signals from Cdc10-YFP (red) and Whi5-GFP (green); the scale bar represents 5 µm.
(3.00 MB AVI)

Video S2 **Response of** ***cln1 cln2 cln3*** **cells to a 10-min-long pulse of exogenous** ***CLN2*** **(corresponding to the data described in** **Figure 3** **).** The left panel shows phase contrast images and cell contours. Color coding indicates cells fate (green contours indicates released cells, whereas red contours indicates blocked cells); The right panel shows Met3pr-Venus and Cdc10-YFP fluorescence signals.
(4.98 MB AVI)

Video S3 **Reversible Start transition in the absence of** ***CLN1,2*** **feedback.** *cln1 cln2 cln3 GAL1pr-SIC1-4A MET3pr-CLN2* cells are shown following two consecutives 15-min-long pulses of exogenous *CLN2* (at *t* = 540 min and *t* = 690 min). The three panels show phase contrast images, *CLN2*pr-Venus-degron signals, and Whi5-GFP signals, respectively. White lines indicate cell contours.
(5.99 MB AVI)

Video S4 **Irreversible Start transition in the presence of** ***CLN1,2*** **feedback.** *cln3 bck2 GAL1pr-SIC1-4A MET3pr-CLN2* cells are shown following a pulse of exogenous *CLN2*. Same legend as Video S3. The 15-min-long pulse is started at *t* = 540 min.
(5.07 MB AVI)

## Abbreviations

WT: wild type
−Met: no methionine

## References

[1] Hartwell L. H, Culotti J, Pringle J. R, Reid B. J (1974). Genetic control of the cell division cycle in yeast.. Science.

[2] Hereford L. M, Hartwell L. H (1974). Sequential gene function in the initiation of Saccharomyces cerevisiae DNA synthesis.. J Mol Biol.

[3] Morgan D. O (2007). The cell cycle: principles of control.

[4] Tyers M, Tokiwa G, Futcher B (1993). Comparison of the Saccharomyces cerevisiae G1 cyclins: Cln3 may be an upstream activator of Cln1, Cln2 and other cyclins.. EMBO J.

[5] Costanzo M, Nishikawa J. L, Tang X, Millman J. S, Schub O (2004). CDK activity antagonizes Whi5, an inhibitor of G1/S transcription in yeast.. Cell.

[6] de Bruin R. A, McDonald W. H, Kalashnikova T. I, Yates J, Wittenberg C (2004). Cln3 activates G1-specific transcription via phosphorylation of the SBF bound repressor Whi5.. Cell.

[7] Oehlen L. J, Cross F. R (1994). G1 cyclins CLN1 and CLN2 repress the mating factor response pathway at Start in the yeast cell cycle.. Genes Dev.

[8] Strickfaden S. C, Winters M. J, Ben-Ari G, Lamson R. E, Tyers M (2007). A mechanism for cell-cycle regulation of MAP kinase signaling in a yeast differentiation pathway.. Cell.

[9] Henchoz S, Chi Y, Catarin B, Herskowitz I, Deshaies R. J (1997). Phosphorylation- and ubiquitin-dependent degradation of the cyclin-dependent kinase inhibitor Far1p in budding yeast.. Genes Dev.

[10] Cross F. R, Tinkelenberg A. H (1991). A potential positive feedback loop controlling CLN1 and CLN2 gene expression at the start of the yeast cell cycle.. Cell.

[11] Dirick L, Nasmyth K (1991). Positive feedback in the activation of G1 cyclins in yeast.. Nature.

[12] Bean J. M, Siggia E. D, Cross F. R (2006). Coherence and timing of cell cycle start examined at single-cell resolution.. Mol Cell.

[13] Dirick L, Böhm T, Nasmyth K (1995). Roles and regulation of Cln-Cdc28 kinases at the start of the cell cycle of Saccharomyces cerevisiae.. EMBO J.

[14] Stuart D, Wittenberg C (1995). CLN3, not positive feedback, determines the timing of CLN2 transcription in cycling cells.. Genes Dev.

[15] Skotheim J. M, Di Talia S, Siggia E. D, Cross F. R (2008). Positive feedback of G1 cyclins ensures coherent cell cycle entry.. Nature.

[16] Xiong W, Ferrell J. E (2003). A positive-feedback-based bistable ‘memory module’ that governs a cell fate decision.. Nature.

[17] Pomerening J. R, Sontag E. D, Ferrell J. E (2003). Building a cell cycle oscillator: hysteresis and bistability in the activation of Cdc2.. Nat Cell Biol.

[18] Pomerening J. R, Kim S. Y, Ferrell J. E (2005). Systems-level dissection of the cell-cycle oscillator: bypassing positive feedback produces damped oscillations.. Cell.

[19] Sha W, Moore J, Chen K, Lassaletta A. D, Yi C (2003). Hysteresis drives cell-cycle transitions in Xenopus laevis egg extracts.. Proc Natl Acad Sci U S A.

[20] Yao G, Lee T. J, Mori S, Nevins J. R, You L (2008). A bistable Rb-E2F switch underlies the restriction point.. Nat Cell Biol.

[21] Amon A, Tyers M, Futcher B, Nasmyth K (1993). Mechanisms that help the yeast cell cycle clock tick: G2 cyclins transcriptionally activate G2 cyclins and repress G1 cyclins.. Cell.

[22] Charvin G, Cross F. R, Siggia E. D (2008). A microfluidic device for temporally controlled gene expression and long-term fluorescent imaging in unperturbed dividing yeast cells.. PLoS ONE.

[23] Verma R, Annan R. S, Huddleston M. J, Carr S. A, Reynard G (1997). Phosphorylation of Sic1p by G1 Cdk required for its degradation and entry into S phase.. Science.

[24] Schneider B. L, Zhang J, Markwardt J, Tokiwa G, Volpe T (2004). Growth rate and cell size modulate the synthesis of, and requirement for, G1-phase cyclins at start.. Mol Cell Biol.

[25] Amon A, Irniger S, Nasmyth K (1994). Closing the cell cycle circle in yeast: G2 cyclin proteolysis initiated at mitosis persists until the activation of G1 cyclins in the next cycle.. Cell.

[26] Lanker S, Valdivieso M. H, Wittenberg C (1996). Rapid degradation of the G1 cyclin Cln2 induced by CDK-dependent phosphorylation.. Science.

[27] Charvin G, Cross F. R, Siggia E. D (2009). Forced periodic expression of G1 cyclins phase-locks the budding yeast cell cycle.. Proc Natl Acad Sci U S A.

[28] Schwob E, Böhm T, Mendenhall M. D, Nasmyth K (1994). The B-type cyclin kinase inhibitor p40SIC1 controls the G1 to S transition in S. cerevisiae.. Cell.

[29] Mateus C, Avery S. V (2000). Destabilized green fluorescent protein for monitoring dynamic changes in yeast gene expression with flow cytometry.. Yeast.

[30] Epstein C. B, Cross F. R (1994). Genes that can bypass the CLN requirement for Saccharomyces cerevisiae cell cycle START.. Mol Cell Biol.

[31] Di Como C. J, Chang H, Arndt K. T (1995). Activation of CLN1 and CLN2 G1 cyclin gene expression by BCK2.. Mol Cell Biol.

[32] McCusker D, Denison C, Anderson S, Egelhofer T. A, Yates J. R (2007). Cdk1 coordinates cell-surface growth with the cell cycle.. Nat Cell Biol.

[33] Di Talia S, Skotheim J. M, Bean J. M, Siggia E. D, Cross F. R (2007). The effects of molecular noise and size control on variability in the budding yeast cell cycle.. Nature.

[34] Tyers M, Tokiwa G, Nash R, Futcher B (1992). The Cln3-Cdc28 kinase complex of S. cerevisiae is regulated by proteolysis and phosphorylation.. EMBO J.

[35] Cross F. R, Blake C. M (1993). The yeast Cln3 protein is an unstable activator of Cdc28.. Mol Cell Biol.

[36] Cross F. R, Archambault V, Miller M, Klovstad M (2002). Testing a mathematical model of the yeast cell cycle.. Mol Biol Cell.

[37] McInerny C. J, Partridge J. F, Mikesell G. E, Creemer D. P, Breeden L. L (1997). A novel Mcm1-dependent element in the SWI4, CLN3, CDC6, and CDC47 promoters activates M/G1-specific transcription.. Genes Dev.

[38] Di Talia S, Wang H, Skotheim J. M, Rosebrock A. P, Futcher B (2009). Daughter-specific transcription factors regulate cell size control in budding yeast.. PLoS Biol.

[39] King R. W, Deshaies R. J, Peters J. M, Kirschner M. W (1996). How proteolysis drives the cell cycle.. Science.

[40] Potapova T. A, Daum J. R, Byrd K. S, Gorbsky G. J (2009). Fine tuning the cell cycle: activation of the Cdk1 inhibitory phosphorylation pathway during mitotic exit.. Mol Biol Cell.

[41] López-Avilés S, Kapuy O, Novák B, Uhlmann F (2009). Irreversibility of mitotic exit is the consequence of systems-level feedback.. Nature.

[42] Nash P, Tang X, Orlicky S, Chen Q, Gertler F. B (2001). Multisite phosphorylation of a CDK inhibitor sets a threshold for the onset of DNA replication.. Nature.

[43] Elowitz M. B, Levine A. J, Siggia E. D, Swain P. S (2002). Stochastic gene expression in a single cell.. Science.

[44] Gillespie D. T (1977). Exact stochastic simulation of coupled chemical reactions.. J Phys Chem.

