# Correction: Origin of Irreversibility of Cell Cycle Start in Budding Yeast

**DOI:** 10.1371/annotation/90916531-1b34-4eb5-92a9-a7f8c5d72318

**Published:** 2011-07-21

**Authors:** Gilles Charvin, Catherine Oikonomou, Eric D. Siggia, Frederick R. Cross

The publication date provided in the footer of the PDF is incorrect. The paper was published in the January 2010 issue. 

