# Supplementary figures and images for: Origin of Irreversibility of Cell Cycle Start in Budding Yeast
Source: PLoS Biol. 2010 Jan 19;8(1):e1000284. doi: 10.1371/journal.pbio.1000284 (PMC2797597; doi:10.1371/journal.pbio.1000284)

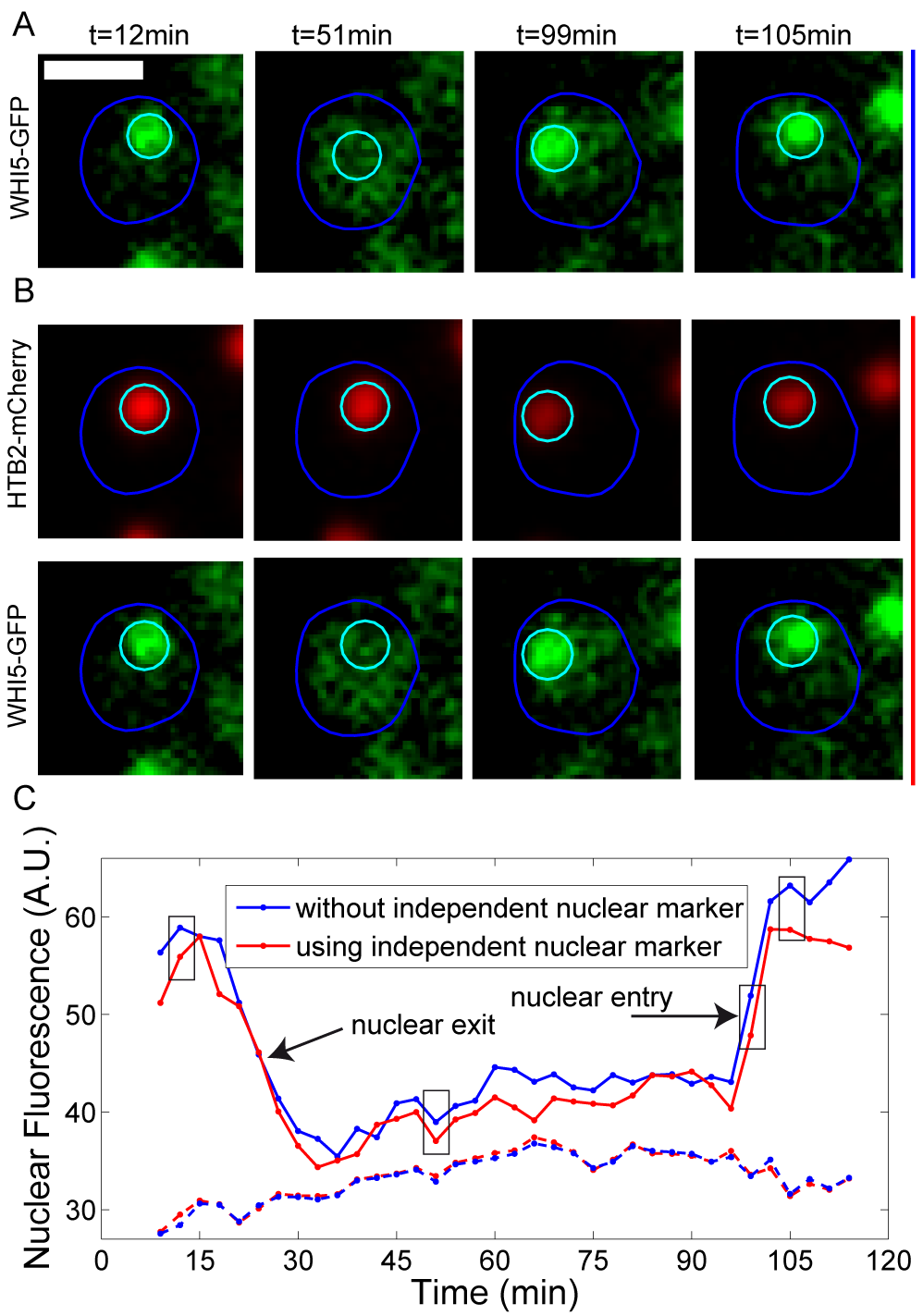

Supplement: Figure S1 — Detection of nuclear fluorescence. (A) Fluorescence images showing the Whi5-GFP in WT cycling cells at indicated timings. The dark blue line indicates the cell contour (retrieved from phase contrast images). The cyan circle shows the position of the nucleus as determined by scoring Whi5-GFP using a custom procedure shown in Text S1. The white rectangle represents 2.5 µm. (B) Same data as in (A), but also displaying the permanent nuclear marker Htb2-mCherry (top images). This marker was used to retrieve the actual position of the nucleus (cyan line), which in turn allowed quantification of the Whi5-GFP nuclear signal (bottom images). (C) Quantification of nuclear signal according to methods described in (A) (blue lines) and (B) (red lines), as a function of time. Solid lines show nuclear signal, whereas dashed line represent cytoplasmic signal. Black rectangles indicate data points shown in (A) and (B). (0.39 MB TIF) [file pbio.1000284.s001.tif]

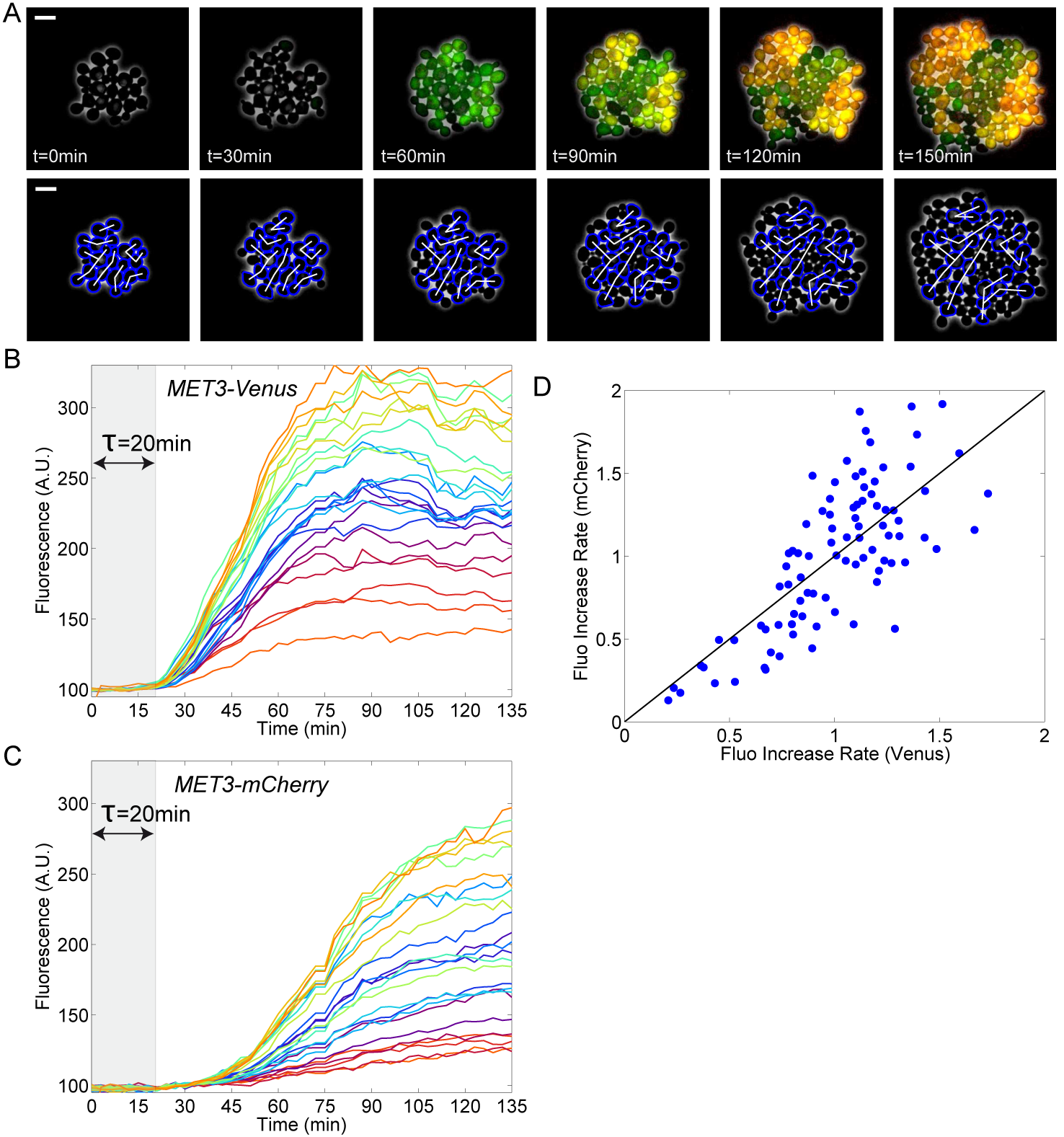

Supplement: Figure S2 — Correlation of expression of MET3-driven fluorophores Venus and Cherry. (A) Top panel: time series of images (overlay of phase plus fluorescence) of a cell colony following a 20-min pulse of −Met. Cells carry MET3-Venus (false colored in green) and MET3-mCherry (colored in red). Bottom panel: phase images as in top panel, plus contours of scored cells (blue lines). White lines indicate cell parentage. (B) MET3-Venus fluorescence trace as a function of time, corresponding to the experiment described in (A). Each colored curve corresponds to a single cell. The shaded area represents the −Met pulse. (C) Same as (B), but for MET3-mCherry. The color coding is consistent with (B). mCherry has a longer maturation half-time than Venus (resp. ∼45 min, unpublished data, versus 18 min, see [22]), thus explaining the observed delay in the rise of fluorescence in (C), as compared to (B). (D) Correlation of transcription rate (as defined by the fluorescence increase rate in [B] and [C]) in the linear part of the curves, and normalized to the mean of each distribution) of the two markers Venus and mCherry over a population of cells. Each blue point corresponds to a single cell. The solid black line is the diagonal. (0.87 MB TIF) [file pbio.1000284.s002.tif]

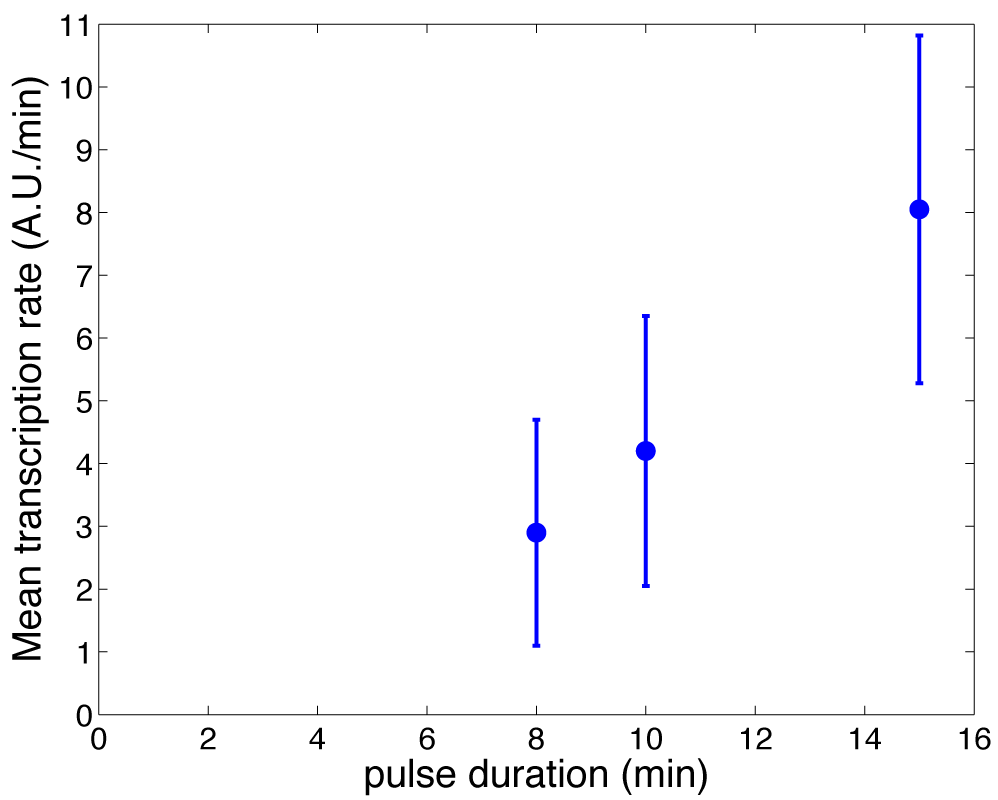

Supplement: Figure S3 — Cell–cell average transcription rate from the MET3pr (MET3pr-Venus construct) as a function of pulse duration (calculated as reported in Figure 3 ). Error bars indicate standard deviation. Each data point shows an average of around 100 cells. (0.06 MB TIF) [file pbio.1000284.s003.tif]

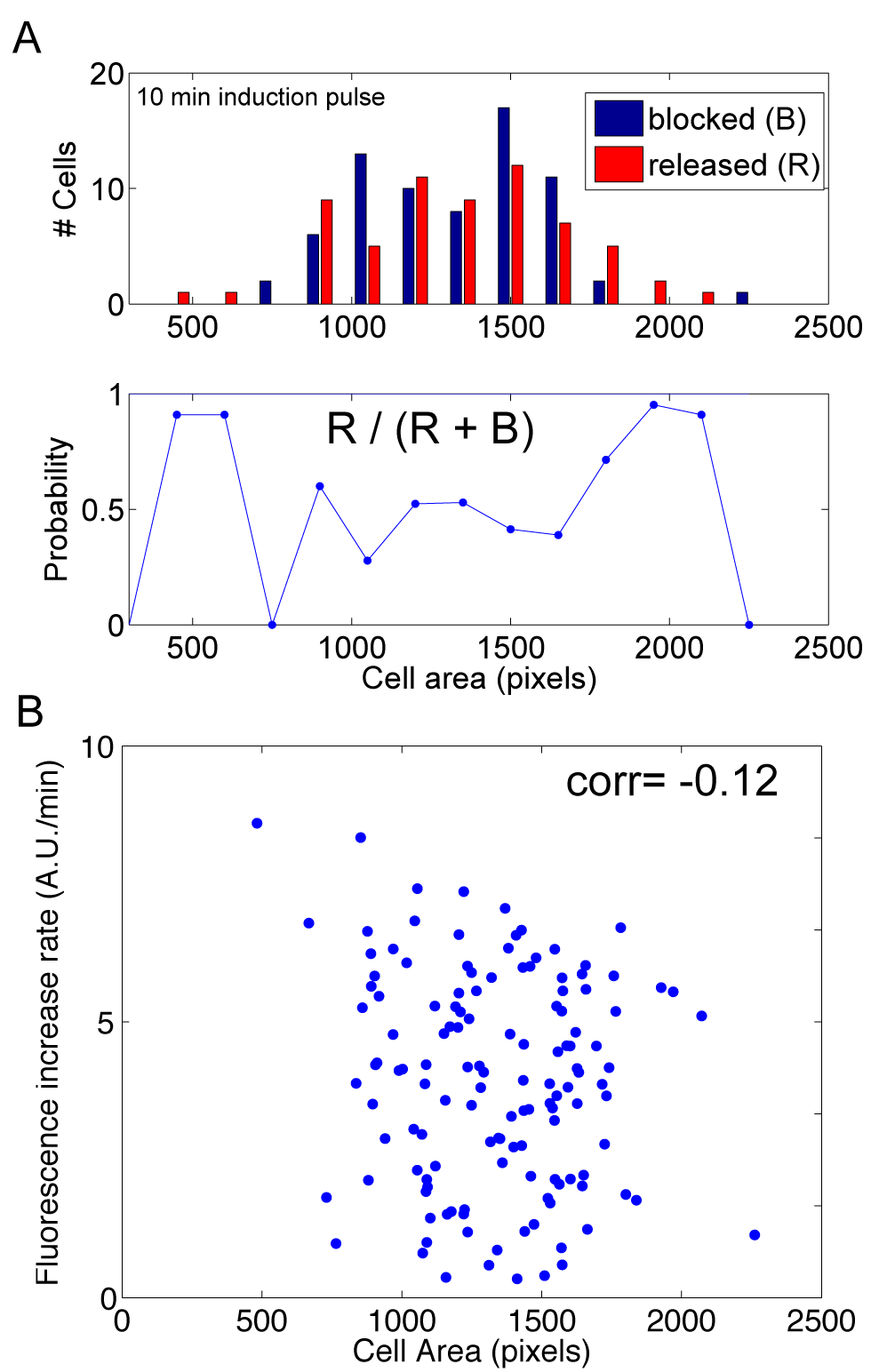

Supplement: Figure S4 — Dependence of Start activation and MET3pr transcription on cell size. (A) Top panel: histogram of cell area (pixels) at division for blocked and released cells, as described in Figure 2F. Bottom panel: probability of passing through Start as a function of cell size. (B) Correlation plot between MET3pr transcription rate and cell area. The coefficient of correlation (corr) is indicated. (0.17 MB TIF) [file pbio.1000284.s004.tif]

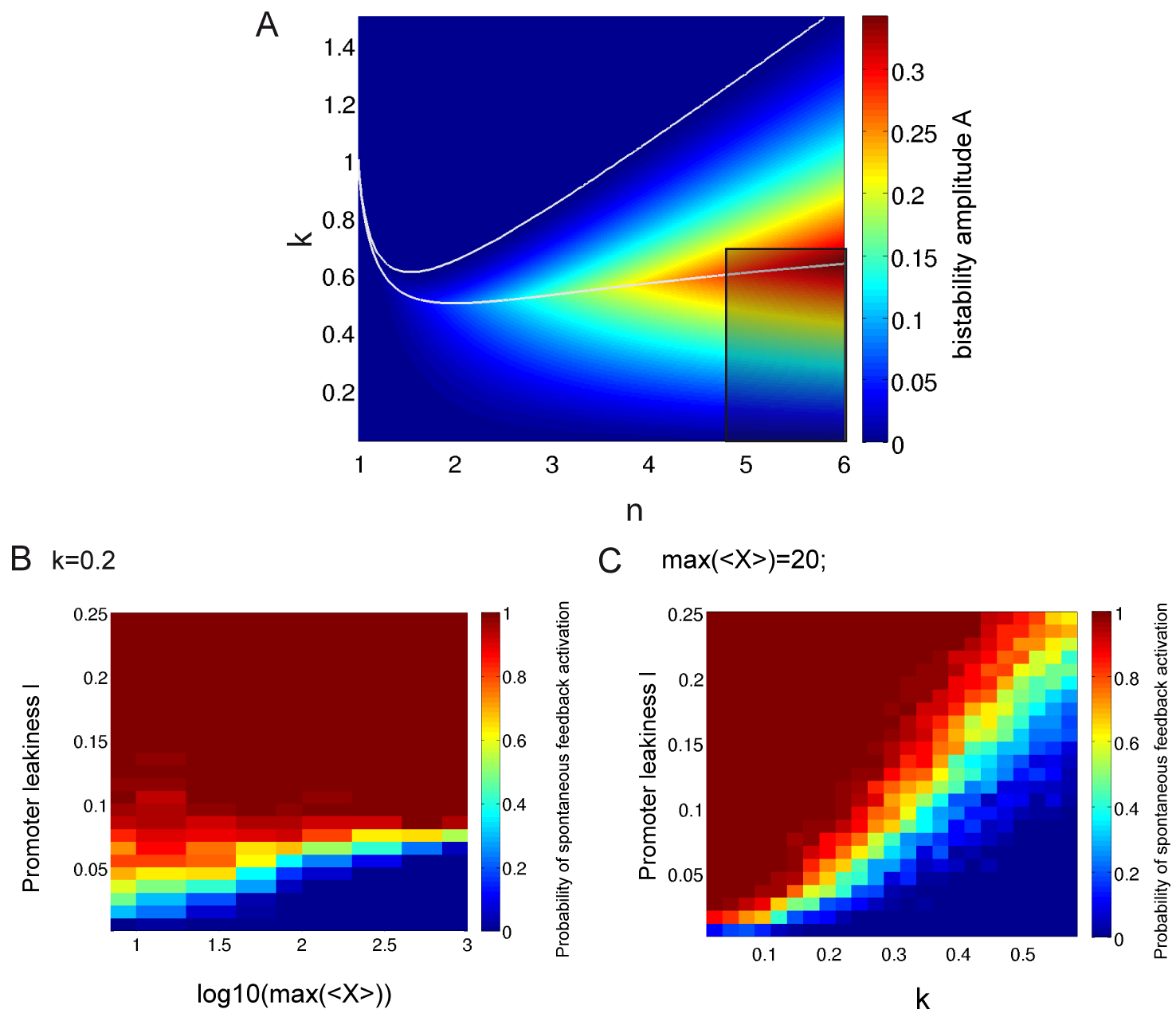

Supplement: Figure S5 — Amplitude of bistability and probability of Start autoactivation as a function of model parameters. (A) Amplitude A of the bistability region as a function of n and k, calculated numerically using the deterministic model described by Equation 2. (B) Probability of observing feedback autoactivation (within 10 units of time) as a function of l and average protein number , using a stochastic version of the model (see Text S1 for details). (C) Probability of feedback autoactivation as a function of k and the leakiness l, using a stochastic simulation of the model. (0.26 MB TIF) [file pbio.1000284.s005.tif]
