# Supplementary material for: Origin of Irreversibility of Cell Cycle Start in Budding Yeast
Source: PLoS Biol. 2010 Jan 19;8(1):e1000284. doi: 10.1371/journal.pbio.1000284 (PMC2797597; doi:10.1371/journal.pbio.1000284)
Supplement: Table S1 — List of strains and plasmids. (0.02 MB DOC) [file pbio.1000284.s006.doc]

Table S1 : List of strains and plasmids used in this study

| **Yeast strains and plasmids used; all derivatives of W303 and originating in this study** | |
| --- | --- |
| **Strain** | **Genotype** |
| GC36-03C | *MATα cln1::HIS3 cln2 cln3::LEU2 TRP1::MET3-CLN2 CDC10-YFP::LEU2 WHI5-GFP::kanMX ade2* |
| CL107 -1 | *MATa cln1::HIS3 cln2::CLN2pr-yEVenus::TRP1 cln3::LEU2 TRP1::MET3-CLN2 GAL1pr-SIC1-4A::URA3 CDC10-YFP::LEU2 WHI5-GFP::kanMX ADE2* |
| CL107-2 | *MATα cln1::HIS3 cln2::CLN2pr-yEVenus-CLN2PEST::TRP1 cln3::LEU2 TRP1::MET3pr-CLN2 CDC10-YFP::LEU2 GAL1pr-SIC1-4A::URA3 ADE2* |
| CL124 | *MATα cln3::LEU2 bck2::HIS3 TRP1::MET3pr-CLN2 GAL1pr-SIC1-4A::URA3 WHI5-GFP::kanMX CDC10-YFP::LEU2 CLN2pr:yEVenus- CLN2PEST::TRP1::CLN2 ADE2* |
| CL146 | *MATα cln1∆ cln2∆ cln3::LEU2 GAL1pr:CLN1::LEU2 URA3::MET3pr-CLN2 TRP1::MET3pr-yEVenus-CLN2PEST ADE2* |
| CL156 | *MATa cln1∆ cln2∆ cln3::LEU2 bck2::HIS3 TRP1::MET3pr-CLN2 WHI5-GFP::kanMX CDC10-YFP::LEU2 ADE2* |
| CL174 | *MATa cln1::HIS3 cln2∆ cln3::LEU2 bck2::HIS3 TRP1::MET3pr-CLN2 GAL1pr-SIC1-4A::URA3 CDC10-YFP::LEU2 WHI5-GFP::kanMX* |
| CL177 | *MATα cln1::HIS3 cln2::CLN2pr-yEVenus::TRP1 cln3::LEU2 TRP1::MET3pr-CLN2 GAL1pr-SIC1-4A::URA3 whi5::kanMX CDC10-YFP::LEU2 ADE2* |
| CL190 | *MATa cln1∆ cln2∆ cln3::LEU2 GAL1pr:CLN1::LEU2 TRP1::MET3pr-CLN2 CDC10-YFP::LEU2 URA3::MET3pr-yEVenus ADE2* |
| CL118 | *MATa MET3:MET3pr-mCherry::URA3::MET3pr-Venus::TRP1 ADE2* |
| GC120 | *MATa HTB2-:mCherry-HIS3 WHI5-GFP::kanMX ADE2* |
| CL142 | *MATa CLN2pr-GFP::HIS3 URA3::GAL-SIC1-4A ADE2* |
| **Plasmid** | **Description** |
| pCL10 | *MET3pr-yEVenus-CLN2_PEST_ ::URA3* |
| pCL17 | *MET3pr-CLN2::URA3* |
| pGC25 | *MET3pr-yEVenus::TRP1* |
| pGC25D | *MET3pr-yEVenus-CLN2_PEST_::TRP1* |
| pCL13 | *MET3pr-mCherry::URA3* |
| pCL25 | *MET3pr-yEVenus::URA3* |
