# Supplementary material for: Origin of Irreversibility of Cell Cycle Start in Budding Yeast
Source: PLoS Biol. 2010 Jan 19;8(1):e1000284. doi: 10.1371/journal.pbio.1000284 (PMC2797597; doi:10.1371/journal.pbio.1000284)
Supplement: Text S1 — Model details and complementary methods. (0.08 MB PDF) [file pbio.1000284.s007.pdf]

Supplementary Information

Gilles Charvin, Catherine Oikonomou, Eric Siggia and Fred Cross

1 Model - determination of the stability map in the (n,k) space

According to the model hypothesis, the equation governing the dynamics of $[Cln2]$ is given by:

$$\frac{d[Cln2]}{dt} = \alpha \frac{[Cln]^n}{[Cln]^n + K^n} - \delta[Cln2] \tag{1}$$

where $[Cln] \equiv [Cln2]+[Cln3]+[Cln2_e]$; $[Cln3]$ is the concentration of the input cyclin Cln3 and $[Cln2_e]$ is the concentration of exogenous Cln2; K is the concentration of cyclin at which Cln2 production has reached its half maximum; n is a Hill coefficient that sets the non-linearity in Cln2 production; δ is the degradation rate of Cln2 and α is the maximum production rate of Cln2.

This system can be rendered dimensionless by normalizing equation (1) by time and maximum [Cln2] concentration, thus leading to the following equation:

$$\frac{dX}{dt} = \frac{(X + X_e)^n}{(X + X_e)^n + k^n} - X \tag{2}$$

where $X_e \equiv \frac{\delta}{\alpha}([Cln3] + [Cln2_e])$ represents the cyclin input, $X \equiv \frac{\delta}{\alpha}[Cln2]$ is the endogenous Cln2, and $k \equiv \frac{\delta}{\alpha}K$.

In order to determine the concentration of $Cln2$ at equilibrium for a given input $Cln3$ or exogenous cyclin $Cln2_e$, one must calculate the fixed points X^* of equation (2), which are such that : $\frac{dX}{dt} = 0$. Therefore, the fixed points X^* are given by:

$$\frac{(X^* + X_e)^n}{(X^* + X_e)^n + k^n} = X^* \tag{3}$$

The aim of the following sections is to determine how the stability curve of X^* as a function of X_e varies in the parameter space n,k. Even though it is in general impossible to explicitly derive the solution $X^* = f(X_e)$, one can derive analytically the conditions on k and n to get a monostable, bistable, and/or irreversible behavior.

1.1 Trivial cases: n=1 or k=0

If k=0, then $X^* = 1$ is the only possible fixed point. In this case, Cln2 is constitutively activated, no matter the value of n.

If n=1, equation (3) can easily be solved and yields one fixed point, which is given by:

$$X^* = \frac{1}{2}\left[(1 - k - X_e) + \sqrt{(X_e + k - 1)^2 + 4X_e}\right] \tag{4}$$

In this case, the expression of the endogenous Cln2 depends on cyclin input in a continuous and monotonous manner.

Therefore, in these two limit cases $n = 1$ (for any k) and $k = 0$ (for any n), the system is monostable. In the following, we therefore consider the cases where $n > 1$ and $k > 0$.

1.2 the bistability/irreversiblity transition

The transition from a bistable to an irreversible system occurs when a branch in the $X^* = f(X_e)$ stability curve crosses the Y-axis (see for instance the third panel in Fig. 7). Indeed, in this case, once the system has reached the activated state, it can no longer switch back to the repressed state: if the input X_e goes back to 0, the activated state is still stable.

Mathematically, irreversiblity thus occurs when $X_e = f^{-1}(X^*) < 0$.
Since f (and f^{-1}) cannot be derived explicitly from eq. (3), we define a parametrization t such that $t = X^* + X_e$. Therefore, X^* and X_e can be rewritten as:

$$X^* = \frac{t^n}{t^n + k^n}$$

and

$$X_e = t - \frac{t^n}{t^n + k^n}$$

X^* varies monotonically with t, so that $X_e = f^{-1}(X^*) < 0$ can be reformulated as the condition for which the minimum of $X_e(t)$ becomes negative.

The value t_{ex} at which $X_e(t)$ reaches an extremum is given by $\frac{dX_e}{dt} = 0$ (and selects the upper branch of X vs X_e since the slope of the sigmoid is greatest there), which yields:

$$(t_{ex}^n + k^n)^2 = n t_{ex}^{n-1} k^n$$

The condition $X_e(t_{ex}) < 0$ can then be rewritten as:

$$k < \frac{1}{n}(n-1)^{\frac{n-1}{n}} \tag{5}$$

1.3 the monostability/bistability transition

The transition from a monostable to a bistable system arises when there is an interval of input cyclin Xe where two stable fixed points co-exist (e.g. k=0.7 and n=4.8 on Fig. 7). Mathematically, this occurs when : $\frac{dX_e}{dX^*} < 0$. Using the same parametrization as above, we calculate the value t_{ex} at which the derivative $\frac{dX_e}{dt}$ is minimal:

$$t_{ex} = k\left(\frac{n-1}{n+1}\right)^{\frac{1}{n}}$$

The condition on k and n can then be written as:

$$k < \frac{1}{4n}(n-1)^{\frac{n-1}{n}}(n+1)^{\frac{n+1}{n}} \tag{6}$$

The two relations (5) and (6) thus partition the (n,k) space into three separate regions, as shown in Fig. 7b.

2 Model - Promoter leakiness and stochastic activation

In order to take into account potential leakiness of the CLN2 promoter, one can rewrite the Cln2 synthesis rate as follows:

$$\text{Cln2 synthesis rate} \equiv \beta \left[l + (1-l) \frac{(X+X_e)^n}{(X+X_e)^n + k'^n} \right]$$

where l $(0 < l < 1)$ represents the basal production rate of Cln2. We also introduce the parameter β in order to set the scale of the mean number of molecules in the activated state in the frame of a stochastic simulation. The typical average number of Cln2 molecules in a fully activated state is $max(< X >) = \beta$. Varying this parameter is useful to determine to what extent stochastic fluctuations in the number of molecules can trigger the auto-activation of this system.

Simulations displayed on Fig. 7e were run according to the standard Gillespie's algorithm (see ref. 45 in the main text), starting with [Cln2]=0 at t=0.

3 Methods - Automated scoring of Whi5-GFP nuclear fluorescence

The total amount of Whi5 within a cell is not cell-cycle regulated (see ref [5] and [6]). Therefore, the sharp increase and decrease of concentrated Whi5-GFP fluorescence within the nucleus is known to be solely due to its shuttling between the nucleus and the cytoplasm (see ref. [5], [6], [12] and [15]). Previous studies have reported the use of an independent nuclear marker (see ref 15) to quantitatively score the amount Whi5-GFP in the nucleus.

In contrast to nuclear Whi5, cytoplasmic Whi5 provides a fluorescence level that is overwhelmed by cell autofluorescence. Therefore, any observed signal that 1) is significantly higher than cellular autofluorescence 2) has the shape and the size of the nucleus can be attributed to the presence of some Whi5 in the nucleus. Therefore, we devised an alternative method of quantitation of nuclear Whi5 using a simple Monte-Carlo approach in the absence of an independent nuclear marker. A comparison to the method that uses an independent marker shows that the techniques are equally effective (see Fig. S1).

3.1 A Monte-Carlo method to detect nuclear fluorescence

Our algorithm assumes that the nucleus is a rounded surface, whose area is around 80-100 square pixels. The localization of the nucleus is determined by letting a circle of a given radius diffuse inside the cell contour (the cell contour is determined previously by segmenting the phase contrast image). The energy of a circle at a given position is dependent upon pixel intensities as follows:

$$E = - \sum_{\text{pixels i in circle}} I_i + k(A - A_0)^2 \tag{7}$$

where I_i is the intensity of the pixel i, A is the area of the circle, A_0 is a reference area and k is a constant that characterizes the strength of control of the nucleus area. Such a definition of energy allows one to look for the region in the cell where intensity of fluorescence is maximal within a circle of variable area A, while allowing the size of the circle to be kept close to a known reference A_0.

A typical try for such optimization was started by randomly choosing the position and size of a circle. At each iteration, the circle was allowed to translate freely inside the contour and to experience a change in radius (increase or decrease). The energy of this new configuration was then recalculated and the new configuration was accepted with a probability p given by:

$$p = min \left[1, \exp \left(-\frac{\Delta E}{kT} \right) \right] \tag{8}$$

where ΔE is the variation of energy between the old and the new configuration and kT is a 'temperature' that characterizes the diffusivity of the circle. 1000 iterations were observed to be largely

sufficient for the circle to localize to the region where the signal was brightest (see left panel in Fig. S1a).

The determination of the position of the nucleus using this method thus allowed us to calculate the mean cytoplasmic fluoresence by substracting the nucleus pixels from the pixels in the whole cell contour. Comparing the obtained intensity of nuclear fluorescence I_n to the cytoplasmic fluorescence I_c revealed that this method is a robust way to score the localization of a transient nuclear marker: $I_n \approx I_c$ indicates that the marker had exited the nucleus (compare solid blue lines to dashed lines in Fig. S1c, where a cycling WT daughter cell was followed over a complete cell cycle), whereas $I_n > I_c$ indicates that the marker is in the nucleus. This could easily be confirmed by visual inspection. In the former case, the displayed position of the circle does not indicate the position of the nucleus, but shows the average of the positions reached during the course of the Monte-Carlo process.

3.2 Comparison to a method that uses an independent nuclear marker

In order to get a better evaluation of the reliability of this method than the visual inspection of the result, we used a test strain that actually carries the permanent nuclear marker Htb2-mCherry (this marker was previously used to score Whi5-GFP, see ref. 15 in the main text) as well as Whi5-GFP. We used our Monte-Carlo procedure to detect the position of the nucleus from the Htb2-mCherry signal (see top panels in Fig. S1b) and then used the found circle to calculate the amount of Whi5-GFP fluorescence.

The agreement between the two methods is remarkable (compare blue and red lines in Fig S1c), thus showing that it the quantitation of the localization Whi5-GFP (and thus its shuttling between the nucleus and the cytoplasm) can be achieved without the need of an independent nuclear marker.

Interestingly, when using an independent marker to locate the nucleus, we noticed a small yet consistent difference between the intensity of nuclear and cytoplasmic GFP fluorescence (compare solid and dashed red lines in Fig. S1c), even when Whi5-GFP was obviously non-nuclear (see t=51min in Fig. S1b). Careful analysis revealed that the level of cytoplasmic cell autofluorescence is usually higher in the middle than on the edges of the cell so that an average over the whole contour is substantially smaller than the average inside a region of the cell that is close to its center.

Last, we observe that the difference between the two methods described above was smaller when scoring cytoplasmic than nuclear fluorescence (compare the differences between red and blue dashed lines versus the red and blue solid lines in Fig. S1c). This effect was due to averaging over a smaller number of pixels in the latter case.
